# An Application of Fit Quality to Screen MDM2/p53 Protein-Protein Interaction Inhibitors

**DOI:** 10.3390/molecules23123174

**Published:** 2018-12-01

**Authors:** Xin Xue, Gang Bao, Hai-Qing Zhang, Ning-Yi Zhao, Yuan Sun, Yue Zhang, Xiao-Long Wang

**Affiliations:** 1 Department of Medicinal Chemistry, Nanjing University of Chinese Medicine, Nanjing 210023, China; 20161291@njucm.edu.cn (G.B.); 20161413@njucm.edu.cn (H.-Q.Z.); 047415246@njucm.edu.cn (Y.Z.); gregwan@njucm.edu.cn (X.-L.W.); 2 Department of Pharmacy, Red Stone Health Industry International Limited, NO.10 Xianlin Street, Nanjing 210038, China; smilezny@126.com; 3 Department of Chemistry and Biochemistry, The Ohio State University, Columbus, OH 43210, USA; sun.596@buckeyemail.osu.edu

**Keywords:** virtual screening, ligand efficiency, fit quality, MDM2/p53 protein-protein interaction

## Abstract

The judicious application of ligand or binding efficiency (LE) metrics, which quantify the molecular properties required to obtain binding affinity for a drug target, is gaining traction in the selection and optimization of fragments, hits and leads. Here we report for the first time the use of LE based metric, fit quality (FQ), in virtual screening (VS) of MDM2/p53 protein-protein interaction inhibitors (PPIIs). Firstly, a Receptor-Ligand pharmacophore model was constructed on multiple MDM2/ligand complex structures to screen the library. The enrichment factor (EF) for screening was calculated based on a decoy set to define the screening threshold. Finally, 1% of the library, 335 compounds, were screened and re-filtered with the FQ metric. According to the statistical results of FQ vs. activity of 156 MDM2/p53 PPIIs extracted from literatures, the cut-off was defined as FQ = 0.8. After the second round of VS, six compounds with the FQ > 0.8 were picked out for assessing their antitumor activity. At the cellular level, the six hits exhibited a good selectivity (larger than 3) against HepG2 (wt-p53) vs. Hep3B (p53 null) cell lines. On the further study, the six hits exhibited an acceptable affinity (range of *K*_i_ from 10^2^ to 10^3^ nM) to MDM2 when comparing to Nutlin-3a. Based on our work, FQ based VS strategy could be applied to discover other PPIIs.

## 1. Introduction

Over the past decade, there has been considerable interest in the discovery of molecules that disrupt the protein-protein interaction (PPI), which play a prominent role in biological processes that are implicated in many diseases. However, the design of novel drug-like small molecule inhibitors of PPIs represents an on-going challenge in current chemical biology. Unlike enzyme or GPCR targets, which have generally yielded to the discovery and design of small molecule drugs, PPIs have been far more difficult targets mainly due to the large, shallow binding surfaces that are frequently involved. Rational virtual screening strategies, like high-throughput screening (HTS), scaffold searching and fragment based drug design (FBDD) are utilized to discover small molecules for disruption of PPIs.

As one of the most important PPIs, MDM2/p53 interaction has long been recognized as a high-risk target due to the fact that proteins generally offered relatively large and flat interacting surfaces. Since the year of 2003 various approaches, like HTS [1,2], pharmacophore screening [3,4,5], dock screening [6], de novo designing [7] and mimicking peptides [8], have been developed and successfully to identify and design MDM2/p53 PPIIs. All these virtual screening (VS) protocols disclosed several lead compounds some of which had promoted into clinical trials like **RG7112** [9], **AM-8553** [10], **RG7388** [11], **AMG 232** [12], **NVP-CGM097** [13], **APG-115** [14] and **SAR405838** [15]. Retrospective analysis of the starting lead-drug pairs from these clinical MDM2 drug discovery programs reveals that the molecular properties (molecular weight and lipophilicity) inflation is very common during lead discovery and optimization; hence there is a need to keep tight control over them during the process of drug evolution [16]. Moreover, the success of developing a drug depends heavily on the nature of the starting hits chosen for lead optimization. Simple criteria such as activity alone is insufficient for selecting an appropriate hit for optimization; additional criteria such as Ligand efficiency (LE) needs to be considered to select the right hit candidates for further optimization [17,18].

LE is defined as free energy or another binding property averaged versus the heavy atom count (HA) [19]. It is used as an early estimator for the potential of drug optimization not only in the context of biological activity but also in its HA, molecular weight (MW) or log P in the drug design process in the pharma industry. On the one hand, it has recently been shown that the LE can be used for efficient modeling of the activity of a compound [20,21]. On the other hand, Sheridan debunked the idea that LE indices are superior to IC_50_ values as quantitative structure−activity relationship alternatives [22]. LE is first introduced in the field of FBDD to select the better one of two fragments and now it is routinely used to choose the optimized leads during hit to drug evolution in drug discovery programs.

The aim of this work is to develop an efficient LE based VS protocol for screening MDM2/p53 PPIIs from the HTS databases. NCI and SPECS databases were prioritized for building an in-house library (supplied by Topscience, Shanghai, China). The Receptor-Ligand pharmacophore model was constructed based on 64 MDM2/ligand complexes and an enrichment factor (EF) was used to define the screening threshold. Successfully 335 compounds were screened to the second round VS using fit quality (FQ). By statistics of 163 reported MDM2/p53 PPIIs, we defined the cut-off as FQ > 0.8. After FQ based VS, 26 compounds with the FQ > 0.8 were purchased for bioassaying. Finally, six hits were validated as potent MDM2/p53 PPIIs with acceptable activity. 

## 2. Results and Discussion

### 2.1. Pharmacophore Model Generation

#### 2.1.1. The Receptor-Ligand Pharmacophore Model Generation

It is known that, ligand-based virtual screening (LBVS), like scaffold-hopping or pharmacophore screening, is much faster than structure-based virtual screening (SBVS), like docking or fragment-based drug design (FBDD). LBVS results in the identification of much more diverse chemotypes and is more useful in scaffold-hopping. In spite of these advantages of LBVS, use of SBVS is often much higher than the use of LBVS methods for hit identification, due to certain limitations [23].

**Figure 1:**
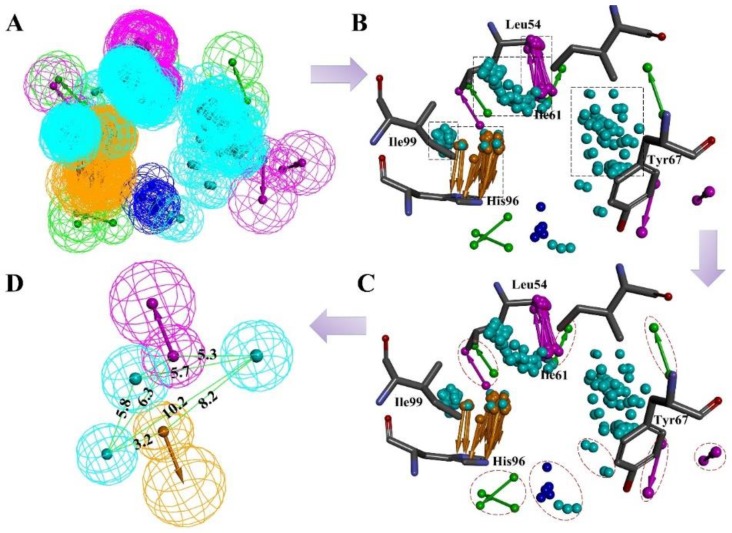
(**A**) The pharmacophore hypotheses were generated by LigandScout algorithm based on 64 MDM2/non-peptide complexes. (**B**) All 64 pharmacophore queries aligned to define the common features. (**C**) All common features were clustered to several parts. The features which unmatched the rule are highlighted by red dotted lines. (**D**) The key features were retained and merged to center features according to the clustering rule. Purple is a hydrogen bond donor; green is a hydrogen bond acceptor, and cyan is a hydrophobic or an aromatic element.

In fact, an ideal pharmacophore model should cover sufficient information about both receptor and ligand. RLPH constructed on receptor/ligand complexes exhibits more information about receptor than ligand based pharmacophore models. Herein, a RLPH was built based on critical interactions of 64 MDM2/non-peptide-inhibitor complexes. The hypothesis was generated using Multi-Protein-Structure (MPS) [3] method which could reflect the protein flexibility. As mentioned above, features generated from 64 complexes were clustered to define the common features (Figure 1A). As was shown in Figure 1B, there were a few features in the center of the binding pocket, while most are distributed around several sub-pockets which contain the critical residues like Leu54, Ile61, Tyr67, His96 and Ile99 of MDM2. Centering on these residues, features were clustered as several sets (Figure 1B). The set that contained less than four features and had a distance longer than 1 Å between its features would be deleted (Figure 1C). According to the clustering rule, these pharmacophore features could be divided into five sets and each set generated one center feature using the Catalyst algorithm. The final hypothesis (Figure 1D and Figure S1, Supplemental Materials) had five features: one AR, one HBD and three HYDs. 

To further confirm that the hypothesis generated was not by chance correlation, EF was calculated using the decoy set containing 156 compounds in test set and 3262 randomly chosen from FDA-approved drugs. The prediction ability of this pharmacophore model was evaluated by the goodness of prediction of MDM2 activity for decoy set compounds. The results are shown in Table S3, Supplemental Materials.

**Figure 2:**
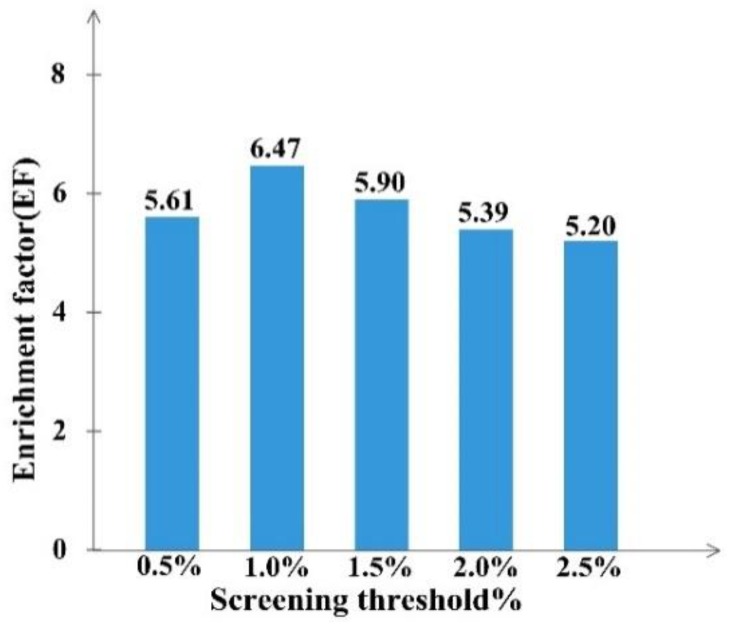
The enrichment factor (EF) at screening threshold of 0.5–2.5% for RLPH screening.

By calculating the EF value in different percent of top molecules ranked with FitValue, we could define the screening threshold of the RLPH, so that the percent of ranked molecules hitting the most active compounds can be confirmed. The results, shown in Figure 2, indicate that the EF value in 0.5%, 1%, 1.5%, 2% and 2.5% top ranked molecules were relatively 5.61, 6.47, 5.90, 5.39 and 5.20. Hit compounds increased along with the total selected molecules. When the percent was greater than 1%, the EF value dropped. Hence, the best percent of ranked molecules to be selected was 1%. 

#### 2.1.2. The Generation and Validation of 3D-QSAR Pharmacophore Model

As an efficient LBVS, 3D-QSAR pharmacophore model is a useful query which exhibits the potent moiety of ligands because the HypoGen algorithm could recognize and distinguish potent inhibitors from weak inhibitors. In this work, 3D-QSAR pharmacophore model is used to predict the activity of screened compounds.

**Figure 3:**
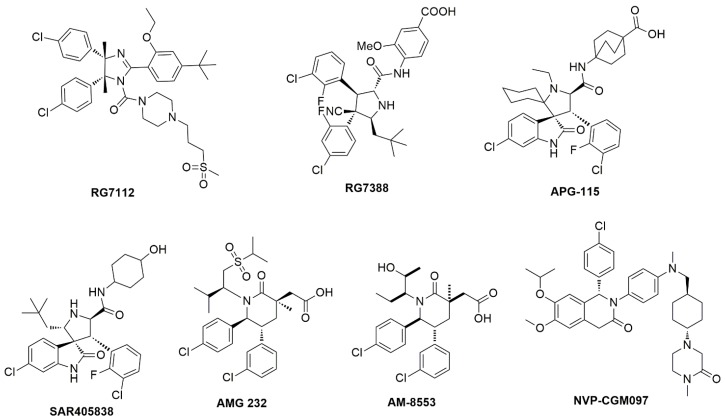
The MDM2/p53 inhibitors which have promoted into clinical trials.

**Figure 4:**
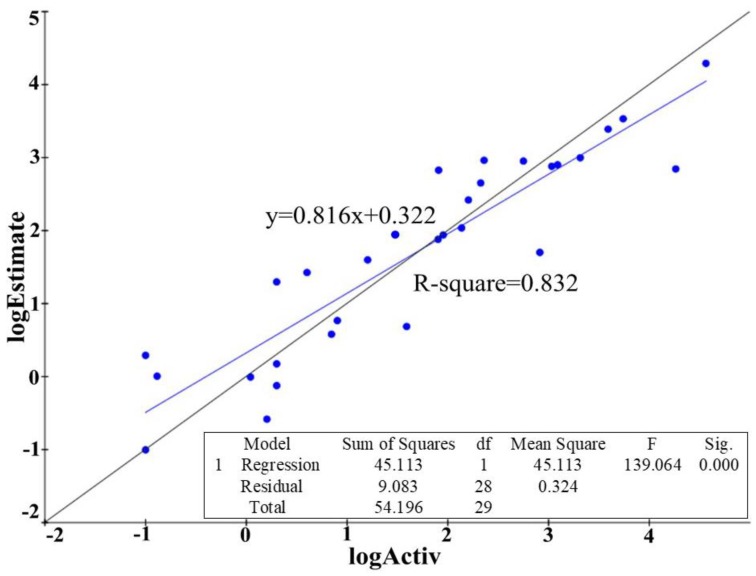
Derivation of r^2^ from the plot of log Activ vs. log Estimate of the best hypothesis built on receptor-ligand complex. The standard deviation is 0.57, root mean square error is 0.912, R-square (r^2^) is 0.832 and the F-statistics is 139.064.

The MDM2/p53 PPIIs in training set used for developing 3D-QSAR pharmacophore model contained eight series of inhibitors (Table S2, Supplemental Materials) together with seven clinical inhibitors (Figure 3). In total 10 hypotheses were generated by the HypoGen algorithm and the hypothesis with the best correlation coefficient was chosen. Derivation of r^2^ from the plot of log Activ vs. log Estimate [24] for the best hypothesis is shown in Figure 4. The r^2^ of the hypothesis was 0.832, close to 1. Different from RLPH, the best 3D-QSAR hypothesis had one hydrogen bond acceptor, one aromatic center and three hydrophobic cores (Figure S3, Supplemental Materials).

### 2.2. Development and Validation of Ligand Efficiency Based Virtual Screening Strategy

After the first step of VS by RLPH, 1% of the library, 335 molecules, were screened with ranked FitValue (Table S3, Supplemental Materials). The screened compounds reveal various molecular sizes, chaotic properties like molecular weight (MW), lipophilicity and number of rings. It became hard work to select potent compounds for bioassaying, though the pharmacophore-based scoring provided an efficient tool to find hundreds of hits among thousands of molecules. 

In the second round of VS, a much more exquisite screening was applied to further inspect those compounds. For this, it was essential for us to develop another cut-off such as Lipinski’s rule of five. Many groups have now used LE and related metrics to assess hits from VS as well as the metrics for optimizing hits in drug discovery programs [25,26]. LE, which is usually defined as the average free energy of binding kcal/mol per non-hydrogen atom (heavy atom or HA), has been employed to explore the essentials of ligand−target binding whereas in our approach, it can be expressed as pIC_50_/HA. Although the use of ligand efficiency indexes in the ranking of virtual screening data is proved to be successful, it cannot be solely used to judge the better one of two hits with disparate size. Analysis of large numbers of protein–ligand complexes over a broad range of affinities [27] demonstrates that average or optimal ligand efficiency (LE) values are systematically higher for small ligands than for large ligands. One size-independent modification of LE using only HA have been proposed: fit quality (FQ). FQ normalizes LE by binning LE values for a large number of disparate complexes and using a scaling factor derived from a spline-fit of the most potent compounds in each bin. In either case, the effect is to transform LE into a metric that is more consistent across broad ranges of molecular size. 

**Figure 5:**
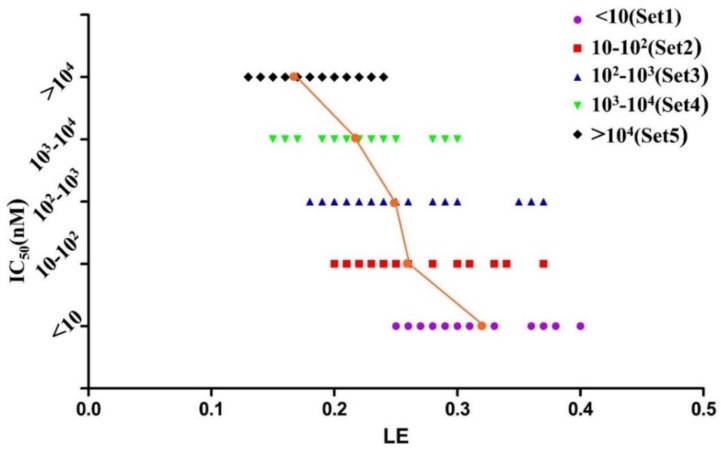
The graph of potency (IC_50_) vs. LE of 156 MDM2/p53 PPIIs.

**Figure 6:**
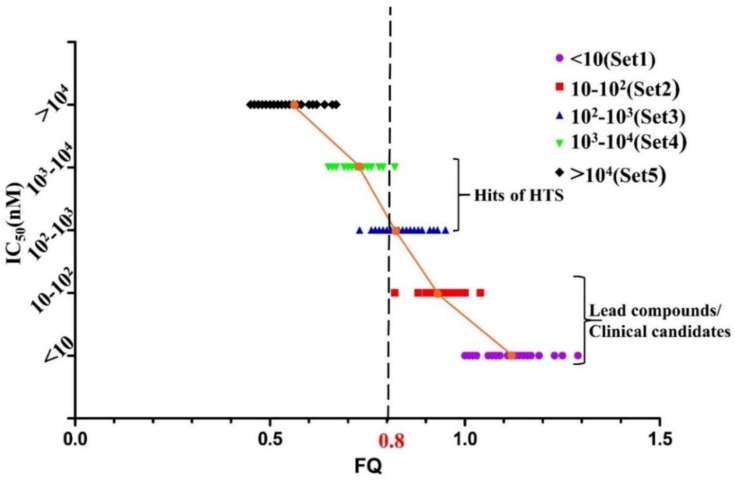
The graph of potency (IC_50_) vs. FQ of 156 MDM2/p53 PPIIs. The cut-off is represented by a black dashed line. The mean value of FQ in Sets 1–5 is relatively 1.11, 0.94, 0.84, 0.73 and 0.55.

The diverse set contains 156 MDM2/p53 PPIIs with eight different cores as well as seven clinical candidates which are also used as the training and test set (Table S2, Group A-H, Supplemental Materials) for constructing the 3D-QSAR pharmacophore model to predict the potency (Estimate, pIC_50_) of screened compounds. The data were collected from the literature and their LE and FQ were calculated. According to their activities, the inhibitors were defined as five sets (Sets 1–5 where the range of IC_50_ values is relatively <10 nM, 10–10^2^ nM, 10^2^–10^3^ nM, 10^3^–10^4^ nM, >10^4^ nM). As shown in Figure 5 and Figure 6, LE ranges from 0.13 to 0.4 while FQ ranges from 0.41 to 1.29. What’s more, the mean LE and FQ values of each set increase as their activities rise. There is no more conclusion can be drawn from Figure 5 except that almost all inhibitors in Set 1 had the LE larger than 0.25 while almost all inhibitors in Set 5 had the LE smaller than 0.25. It remains difficult to define a cut-off using LE since there is no obvious boundary around LE between Set 2–Set 4. 

On the contrary, the boundary of FQ between each set is relatively much clearer in Figure 6. Since the FQ value of each set tends to be more convergent, it is easier to differentiate active compounds from inactive compounds with their FQ values. The conclusion can be drawn easily that the smallest FQ value of inhibitors in Set 1 and Set 2 are relatively 1.0 and 0.8. In addition, the mean value of FQ in Set 4 is 0.73 which is larger than the largest FQ value in Set 5. Interestingly, the range of FQ overlaps a lot between Set 2, Set 3 and Set 4, meanwhile the mean value of FQ for Set 3 and Set 4 is 0.8. It is obvious that the range of activities for compounds at different stages during VS and hit-to-lead study can be defined to be 10^2^–10^4^ nM for hits of HTS and <10^2^ nM for lead or clinical candidates. In order to differentiate active hits from inactive hits and acquire potent compounds as far as possible, the cut-off can be defined as FQ > 0.8. At this point, compounds in Set 5 will barely be screened while half of the compounds in Set 3 and Set 4 will be chosen as hits. It is the balance between low false positive rate and molecular diversity in FQ-based VS.

### 2.3. Hit Identification

**Figure 7:**
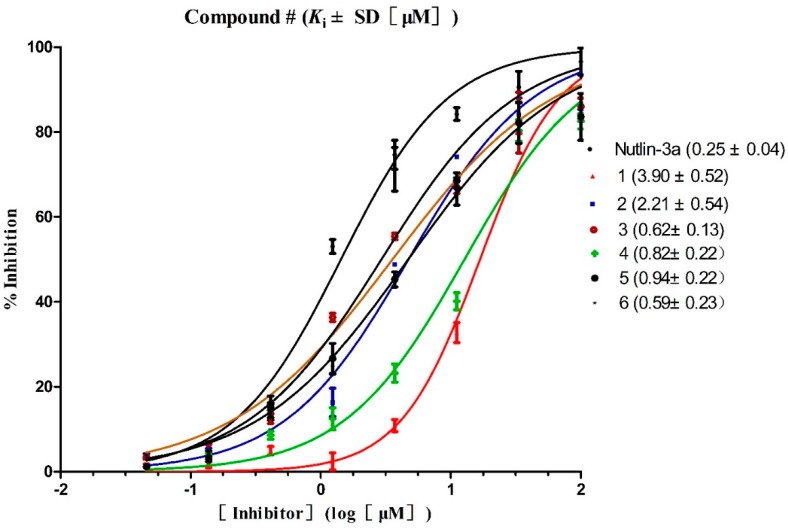
Competitive binding curves of small-molecule inhibitors to MDM2/p53 PPI as determined using a fluorescence-polarization-based binding assay.

Three hundred and thirty-five (335) compounds screened by RLPH were re-ranked with the FQ metrics. Out of the 335 compounds, 26 compounds with the FQ larger than 0.8 were subjected to bioassay (fluorescence polarization competing assay; FP). A sensitive and quantitative FP-based binding assay using human recombinant His-fused soluble protein MDM2 (residues 1–118) and a p53-based peptide labeled with a fluorescence tag, termed as PMDM6-F (10 nM; Anaspec, Fremont, CA 94555, USA). Out of the 26 compounds tested, six compounds, **1**–**6**, showed medium potency (Figure 7). In order to further confirm whether they inhibit MDM2 and release p53, a cellular test using HepG2 cell line (wild-type p53; wt-p53) vs. Hep3B cell line (p53 null) was carried out. The selectivity was calculated by IC_50_ against Hep3B divided by HepG2 (Table 1). The larger the selectivity value was, the stronger the interaction force between the ligands and MDM2.

*K*_i_ values of the six compounds range from 10^2^ nM to 10^3^ nM. Four compounds (**3**, **4**, **5** and **6**) possess potential inhibitory similar to **Nutlin-3a**, while two compounds, **1** and **2**, possess weaker potency, *K*_i_ =3.90 ± 2.60 μM and 2.21 ± 0.54 μM. Interestingly, all six compounds show medium inhibitory activity against the HepG2 cell line. The selectivity of each compound is higher than 1. The highest selectivity among the six compounds is displayed by compound **3** with a value (10.75) similar to **Nutlin-3a** (10.97) and is definitely targeted to disrupt MDM2/p53 PPI at cellular level. The result reminds us that compound **3** shows equal effects to **Nutlin-3a** both at both the cellular and the protein level. 

**Figure 8:**
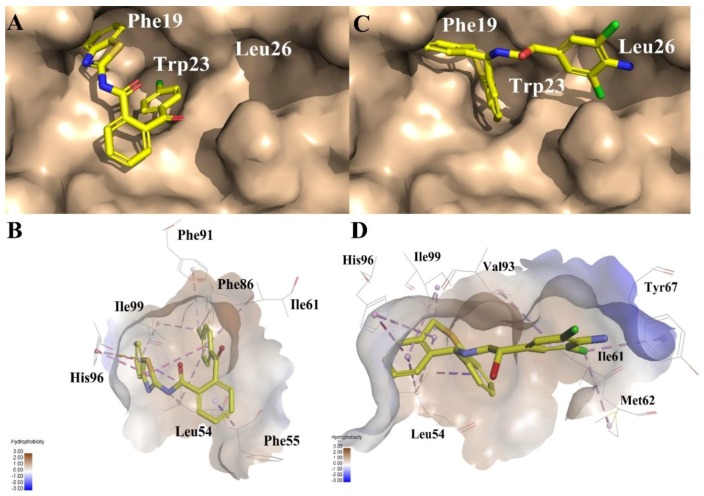
Predicted binding modes of (**A**) and (**B**) compound **3**, (**C**) and (**D**) compound **4** to MDM2. The protein displays as α helix with a gray surface. The HB donor and acceptor are displayed as purple and green. All compounds are shown with only backbone atoms.

To evaluate their ability of binding to MDM2, we placed compounds into the MDM2 pocket by using an exquisite docking program to observe the interactions between the receptor and ligands. Compound **3** obtained the highest docking score and occupied a similar pose as **Nutlin-3a** (the binding mode of **Nutlin-3a** to MDM2 can be found in the Supplemental Materials). As was shown in Figure 8A, the two sub-pockets, pocket Phe19 and Trp23, were occupied by benzothiazole and chlorobenzene moiety of compound **3** and where π-π interactions were formed between MDM2 His96, Phe86 and compound **3**. In Figure 8C, the two sub-pockets were taken up by benzene groups of compound **4** while the Leu26 sub-pocket was taken up by a substituted benzene moiety. Only one π-π interaction was formed between compound **4** and MDM2 His96. Different from compound **4**, compound **3** had a much more flexible scaffold resulting in a docking mode similar to that of **Nutlin-3a**. 

**Figure 9:**
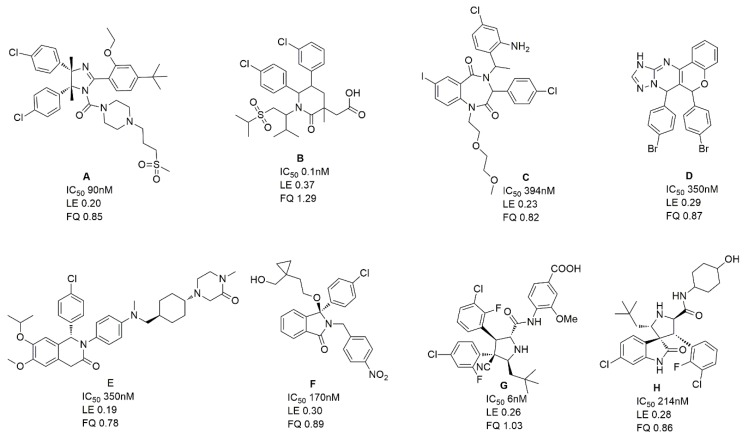
The chemical structures, LE and FQ of eight groups of MDM2/P53 PPIIs used in this study.

Compound **4** is dihydrodibenzo analogue which is similar to compound **C** in Figure 9. Compound **1**, with the largest FQ among the six compounds, possesses the same indolone scaffold as the clinical candidate **APG-115** (Figure 3). Both compounds **2** and **5** have the same moiety, a benzenesulfonamide group. Probably because only two rings exist in compounds **1** and **2**, or the molecular size of the two compounds is small, their activities are weaker than those of the other four compounds. However, it is apparent that small molecules could be easily modified for further study. Structure derivation and synthesis could be focused on the three functional groups, pocket Phe19, Trp23 and Leu26, to further enhance the potency. 

## 3. Conclusions

The search of modulators of protein-protein interactions (PPIs) is currently a challenging issue due to the relatively large interaction surfaces. Hence, identifying a suitable PPII for further optimization to a lead in the early stages of drug discovery is an important step. Virtual screening (VS) is extensively used to identify suitable PPI hits and strategies to improve the VS hit rate (% of hits identified to compounds tested biochemically) are complementary to other approaches. Herein, a ligand efficiency (LE)-based metric, fit quality (FQ), is utilized to rank database compounds and prioritize them for MDM2/p53 PPIIs bioassays. 

A receptor-ligand pharmacophore model was ensembled from 64 MDM2/ligand complexes to screen the library built on NCI and SPECS databases. An enrichment factor (EF) was used to define the screening threshold as 1%. Out of 36,159 molecules, 335 compounds were picked with the ranked FitValue. Then pIC_50_ values were predicted by a 3D-QSAR pharmacophore model to calculate the FQ to re-rank these compounds. According to the analysis of a test set of 156 MDM2/p53 PPIIs, the cut off was defined as FQ > 0.8. Twenty six (26) hits with the FQ larger than 0.8 were purchased to test their inhibitory activity towards MDM2. Finally, six compounds were selected with definite acceptable potency similar to **Nutlin-3a** both at the cellular level and protein level. Subsequent efforts will be made to discover the details when they bind to MDM2 so as to modify the hits to improve their potency. In addition to the success with chemical diversity, the hit rate is nearly 23%. This demonstrates the ability of our FQ-based VS strategy to effectively search and identify more diverse PPIIs.

## 4. Materials and Methods

### 4.1. Pharmacophore Generation

#### 4.1.1. The Generation and Validation of Receptor-Ligand Pharmacophore Model

As was previously reported [28], the pharmacophore model for VS was constructed from receptor-ligand based pharmacophore models (RLPH) which pay more attention to interactions between MDM2 and ligands than that built on ligands. Sixty four (64) MDM2/ligand complex structures reported to date (Table S1, Supplemental Materials) were aligned and prioritized for constructing RLPH. Most of their native ligands or their derivatives, as shown in Figure 8, were recently reported to be advanced into clinical trials.

The pharmacophore hypotheses were constructed using the LigandScout [29] algorithm with the Receptor-Ligand Pharmacophore Generation module within Discovery Studio 4.0 (DS). The LigandScout algorithm allowed the automatic construction of the pharmacophore model from the structural data of the protein-ligand complex. These pharmacophore queries focused on three critical points located by Phe19, Trp23 and Leu26 of p53 in the binding pocket. Sixty four (64) initial queries with their features were aligned and clustered using the Catalyst algorithm to define the center feature. Finally, a pharmacophore hypothesis based on multiple receptor-ligand complex structures were generated for VS.

The enrichment of decoy ligands among top ranked compounds was an important criterion for measuring the credibility of a pharmacophore-based screening. The quality of the pharmacophore hypothesis constructed and the screening threshold for VS by RLPH can be assessed by the Fischer validation which is based on Fischer’s randomization test by randomly assigning the activities of the training set molecules as decoys. One hundred and fifty six (156) MDM2/p53 PPIIs with inhibitory activity ranging from 10^−1^ nM to 10^5^ nM collected from the literature and 3262 FDA-approved drugs (downloaded from the ZINC database) formed the decoy database. The enrichment factor (EF) [30] was calculated using the following formula:

(1) $$\mathrm{EF}=\frac{a/n}{A/N}$$

where, *n* = number of the compounds in the top x% of the database, *a* = the number of active ligands present in the top x% of the database, *N* = total number of molecules in database, and *A* = the total number of actives in the database.

#### 4.1.2. The Generation 3D-QSAR Pharmacophore Model

The 156 MDM2/p53 PPIIs divided into eight groups (Group A-H) extracted from references and including seven clinical candidates were assigned to the training and test sets (Table S2, Supplemental Materials). The 3D-QSAR pharmacophore model was generated from molecules in training set by employing 3D QSAR Pharmacophore Generation module within DS. The activities (IC_50_ values) of compounds ranged from 10^−1^ nM to 10^5^ nM. Minimum interfeature distance was set as 3.0 Å because MDM2/p53 PPIIs were usually medium or large molecules. Minimum and maximum pharmacophore features were set as 3 and 5, respectively. Four pharmacophore features were derived by the HypoGen algorithm: hydrogen bond acceptor (HBA), hydrogen bond donor (HBD), aromatic center (AR), and hydrophobic core (HYD).

### 4.2. Virtual Screening 

#### 4.2.1. Pharmacophore Screening

The first round of VS was done with RLPH by using Ligand Pharmacophore Mapping module within DS. The RLPH was utilized to screen an in-house library containing 36,159 (21,235 + 14,924) molecules from the NCI Plated 2007 Database and SPECS Database. The library was built of multi-conformers by using the ‘Build 3D Database’ module in DS (best method, maximum number of conformers = 255). The multi-conformers were generated for the VS by employing the RLPH using Search 3D Database module with the fast flexible search method in DS. The index ‘FitValue’ was calculated to rank the screened molecules. Conformers belonged to the same molecule were also ranked and the best conformer with the highest FitValue remained.

#### 4.2.2. FQ-based Screening

The second round was carried out based on FQ. All the molecules which passed the RLPH screening were aligned to acquire their predictive potency, pIC_50_, by employing the 3D-QSAR pharmacophore model with the Ligand Pharmacophore Mapping module within DS. The heavy atoms (HA) of the compounds were counted and then, LE and FQ values of each molecule were calculated using the following formulas [19]:

LE = −2.303(RT/HA) × logK_d_ = (1.37/HA) × pIC_50_ (2)

FQ = [pIC_50_ ÷ HA] ÷ [0.0715 + (7.5328 ÷ HA) + (25.7079 ÷ HA^2^) − (361.4722 ÷ HA^3^)] (3)

### 4.3. Bioassay

#### 4.3.1. In Vitro Antitumor Activity

The cellular growth inhibitory activity was determined using two human hepatoma cell lines, HepG2 (with wild-type p53) and Hep3B (with wt-p53 null). The IC_50_ was then analyzed using the GraphPad Prism 5 software (see Supplemental Materials).

#### 4.3.2. Fluorescence Polarization Binding Assay

The compounds identified as possible MDM2 inhibitors were purchased from Topscience, Shanghai, China. For testing their binding affinities to MDM2 protein, we performed a sensitive and quantitative FP-based binding assay [3] using human recombinant GST-tag protein MDM2 (residues 1–118) and a p53-based peptide labeled with a fluorescence tag, termed as PMDM6-F (Anaspec, 10 nM). Binding constant (*K*_i_) and inhibition curves were fitted using GraphPad Prism 5 software and a web-based computer program developed by Wang [31].

## Figures and Tables

**Table 1:** Binding constants (*K*_i_) of the two compounds and IC_50_ values of in vitro antitumor activity.

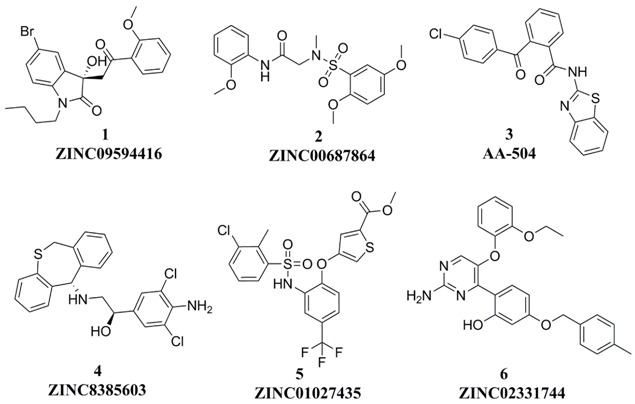						
	***K*_i_^a^ (μM)**	**MTT ^b^ IC_50_^c^ (μM)**		**Selectivity ^d^**	**FQ ^e^**	**FQ-Test ^f^**
		**HepG2 (wt-p53)**	**Hep3B (p53 null)**			
**1**	3.90	8.63	77.51	8.98	1.00	0.75
**2**	2.21	5.38	33.51	6.23	0.85	0.78
**3**	0.62	4.40	47.29	10.75	1.16	0.86
**4**	0.82	5.35	34.81	6.51	0.98	0.83
**5**	0.94	4.77	23.21	4.87	0.96	0.80
**6**	0.59	5.54	20.53	3.71	0.98	0.83
**Nutlin-3a**	0.25	2.31	25.35	10.97	1.01	0.84

^a^ Values are determined by fluorescence polarization assay. ^b^ Values are means of three experiments. ^c^ IC_50_, compound concentration required to inhibit tumor cell proliferation by 50%. ^d^ Selectivity is calculated by IC_50_ of Hep3B (p53 null) divided by HepG2 (wt-p53). ^e^ FQ is calculated by using predicted pIC_50_ estimated by 3D-QSAR pharmacophore model. ^f^ FQ-test is calculated by using p*K*_i_ tested by FP.

## Supplementary Materials

The following are available online. Figure S1: The crystal complexes for Receptor-Ligand pharmacophore model generation. Figure S2: (**A**) The detail information for 3D-QSAR pharmacophore model. (**B**) The clinical candidate, AMG232, was docked into the 3D-QSAR pharmacophore model. Figures S3–S24: The chemical structures, the curve of inhibitory and the binding modes of the six hits. Table S1: The detail information for Receptor-Ligand pharmacophore model. Table S2: Eight groups of MDM2/p53 PPIIs were collected for training and test set. Table S3: Validation results of the hit identification rate (higher EF) at all levels of screening threshold for constructed Receptor-Ligand pharmacophore model. Table S4: 1% out of the library, 335 compounds, passed the first VS by Receptor-Ligand pharmacophore model from NCI and SPECS databases. Table S5: All 26 compounds with the ranked FQ values (FQ > 0.8) were screened in the second VS. Result S1: 1T4E-Docking results. Result S2: 3D-QSAR results.
