# Supplementary material for: An Application of Fit Quality to Screen MDM2/p53 Protein-Protein Interaction Inhibitors
Source: Molecules. 2018 Dec 1;23(12):3174. doi: 10.3390/molecules23123174 (PMC6321222; doi:10.3390/molecules23123174)
Supplement: Supplementary file 1 [file molecules-23-03174-s001.zip › supplemental files/3D-QSAR result.html]

1. Summary of Run Parameters:

|  |  |  |  |  |  |  |  |  |  |  |  |  |  |  |  |  |  |  |  |  |  |  |  |  |  |  |  |  |  |  |  |  |  |
| --- | --- | --- | --- | --- | --- | --- | --- | --- | --- | --- | --- | --- | --- | --- | --- | --- | --- | --- | --- | --- | --- | --- | --- | --- | --- | --- | --- | --- | --- | --- | --- | --- | --- |
| |  |  | | --- | --- | | HypoGen Parameters | | | Spacing | 300 | | Variable Weight | No | | Variable Tolerance | No | | |  |  |  | | --- | --- | --- | | Features Constraints | | | | Name | Min | Max | | HBA | 0 | 5 | | HBD | 0 | 5 | | HYDROPHOBIC | 0 | 5 | | PosIonizable | 0 | 5 | | RING\_AROMATIC | 0 | 5 | | Total | 1 | 5 | |

2. Overall Results:

|  |  |  |  |  |  |  |  |  |  |  |  |  |  |  |  |  |  |  |  |  |  |  |  |  |  |  |  |  |  |  |
| --- | --- | --- | --- | --- | --- | --- | --- | --- | --- | --- | --- | --- | --- | --- | --- | --- | --- | --- | --- | --- | --- | --- | --- | --- | --- | --- | --- | --- | --- | --- |
| |  | | --- | | Pharmacophore Space: 7.109556e+005 | | Best records in pass: 6. | | Fixed Cost: 91.5695 | | Null Cost: 1164.49 | | |  |  | | --- | --- | | Cost Analysis (Fixed/Null distance = 1072.9 bits) | | | Index | Null Cost Distance | | 1 | 871.87 | | 2 | 773.78 | | 3 | 736.32 | | 4 | 729.46 | | 5 | 728.01 | | 6 | 725.15 | | 7 | 716.44 | | 8 | 704.38 | | 9 | 699.49 | | 10 | 695.78 | | |  |

3. Hypotheses Results:

Hypothesis 1

|  |  |  |  |  |  |  |  |  |  |  |  |  |  |  |  |  |  |  |  |  |  |  |  |  |  |  |  |  |  |  |  |  |  |  |  |  |  |  |  |  |  |  |  |  |  |  |  |  |  |  |  |  |  |  |  |  |  |  |  |  |  |  |  |  |  |  |  |  |  |  |  |  |  |  |  |  |  |  |  |  |  |  |  |  |  |  |  |  |  |  |  |  |  |  |  |  |  |  |  |  |  |  |  |  |  |  |  |  |  |  |  |  |  |  |  |  |  |  |  |  |  |  |  |  |  |  |  |  |  |  |  |  |  |  |  |  |  |  |  |  |  |  |  |  |  |  |  |  |  |  |  |  |  |  |  |  |  |  |  |  |  |  |  |  |  |  |  |  |  |  |  |  |  |  |  |  |  |  |  |  |  |  |  |  |  |  |  |  |  |  |  |  |  |  |  |  |  |  |  |  |  |  |  |  |  |  |  |  |  |  |  |  |  |  |  |  |  |  |  |  |  |  |  |  |  |  |  |  |  |  |  |  |  |  |  |  |  |  |
| --- | --- | --- | --- | --- | --- | --- | --- | --- | --- | --- | --- | --- | --- | --- | --- | --- | --- | --- | --- | --- | --- | --- | --- | --- | --- | --- | --- | --- | --- | --- | --- | --- | --- | --- | --- | --- | --- | --- | --- | --- | --- | --- | --- | --- | --- | --- | --- | --- | --- | --- | --- | --- | --- | --- | --- | --- | --- | --- | --- | --- | --- | --- | --- | --- | --- | --- | --- | --- | --- | --- | --- | --- | --- | --- | --- | --- | --- | --- | --- | --- | --- | --- | --- | --- | --- | --- | --- | --- | --- | --- | --- | --- | --- | --- | --- | --- | --- | --- | --- | --- | --- | --- | --- | --- | --- | --- | --- | --- | --- | --- | --- | --- | --- | --- | --- | --- | --- | --- | --- | --- | --- | --- | --- | --- | --- | --- | --- | --- | --- | --- | --- | --- | --- | --- | --- | --- | --- | --- | --- | --- | --- | --- | --- | --- | --- | --- | --- | --- | --- | --- | --- | --- | --- | --- | --- | --- | --- | --- | --- | --- | --- | --- | --- | --- | --- | --- | --- | --- | --- | --- | --- | --- | --- | --- | --- | --- | --- | --- | --- | --- | --- | --- | --- | --- | --- | --- | --- | --- | --- | --- | --- | --- | --- | --- | --- | --- | --- | --- | --- | --- | --- | --- | --- | --- | --- | --- | --- | --- | --- | --- | --- | --- | --- | --- | --- | --- | --- | --- | --- | --- | --- | --- | --- | --- | --- | --- | --- | --- | --- | --- | --- | --- | --- | --- | --- | --- | --- | --- |
| |  |  |  |  |  |  |  |  |  |  |  |  |  |  |  |  |  |  |  | | --- | --- | --- | --- | --- | --- | --- | --- | --- | --- | --- | --- | --- | --- | --- | --- | --- | --- | --- | | |  |  |  |  |  |  |  |  |  |  |  |  |  |  |  |  |  |  | | --- | --- | --- | --- | --- | --- | --- | --- | --- | --- | --- | --- | --- | --- | --- | --- | --- | --- | | |  |  | | --- | --- | | Results | | | Maximum Fit | 19.1533 | | Total Cost | 292.62 | | RMS | 3.48977 | | Correlation | 0.912868 | | |  |  | | --- | --- | | Description | | | Features | Weights | | HBA  HYDROPHOBIC  HYDROPHOBIC  HYDROPHOBIC  RING\_AROMATIC | 3.83066  3.83066  3.83066  3.83066  3.83066 | | | |  | | |  |  |  |  |  |  |  | | --- | --- | --- | --- | --- | --- | --- | | Name | Fit | Est | Act | Err | Status | Mapping | | 4OAS | 14.010 | 1.9000 | 0.1 | 19 | active | [17 3 21 \* 24 ] | | 4QOC\_35T\_C\_201 | 15.300 | 0.098000 | 0.1 | -1 | active | [17 3 39 34 22 ] | | 5LN2 | 14.330 | 0.92000 | 0.13 | 7.1000 | active | [\* 15 7 6 34 ] | | 4erf | 14.330 | 0.92000 | 1.1 | -1.2000 | moderately active | [17 3 21 \* 26 ] | | 4ZTI | 14.850 | 0.28000 | 1.6 | -5.8000 | moderately active | [30 31 18 36 1 ] | | 4OCC | 14.100 | 1.6000 | 2 | -1.3000 | moderately active | [16 3 33 \* 21 ] | | Molecule1 | 13.030 | 18 | 2 | 9.2000 | moderately active | [23 30 25 28 \* ] | | Molecule2 | 14.410 | 0.77000 | 2 | -2.6000 | moderately active | [43 34 39 20 \* ] | | Molecule3 | 12.870 | 26 | 4 | 6.6000 | moderately active | [39 30 \* 24 15 ] | | 4LWV | 13.730 | 3.7000 | 7 | -1.9000 | moderately active | [\* 30 4 11 19 ] | | 4OQ3 | 13.530 | 5.8000 | 8 | -1.4000 | moderately active | [23 30 18 \* 2 ] | | Molecule4 | 12.630 | 46 | 16 | 2.9000 | moderately active | [\* 24 31 32 15 ] | | 4DIJ | 12.410 | 77 | 30 | 2.6000 | moderately active | [15 3 20 \* 2 ] | | Molecule5 | 13.640 | 4.5000 | 39 | -8.7000 | moderately active | [\* 27 34 40 15 ] | | 1t4e | 12.460 | 67 | 80 | -1.2000 | moderately active | [24 18 29 1 \* ] | | Molecule6 | 11.470 | 660 | 81 | 8.2000 | moderately active | [\* 36 21 24 \* ] | | 4HGF | 12.410 | 76 | 90 | -1.2000 | moderately active | [6 14 27 38 \* ] | | Molecule7 | 12.270 | 100 | 140 | -1.3000 | moderately active | [25 \* 12 33 15 ] | | 1TTV | 11.920 | 240 | 160 | 1.5000 | moderately active | [\* 19 27 24 6 ] | | Molecule8 | 11.630 | 460 | 210 | 2.2000 | moderately active | [24 26 12 33 \* ] | | Molecule9 | 11.330 | 920 | 230 | 4 | moderately active | [\* 24 \* 31 15 ] | | Molecule10 | 11.340 | 900 | 560 | 1.6000 | inactive | [\* 21 \* 29 12 ] | | Molecule11 | 12.590 | 51 | 820 | -16 | inactive | [30 1 33 27 \* ] | | Molecule12 | 11.410 | 770 | 1100 | -1.4000 | inactive | [\* 21 \* 28 12 ] | | 3JZK | 11.390 | 790 | 1200 | -1.5000 | inactive | [\* 22 7 4 \* ] | | Molecule13 | 11.290 | 1000 | 2100 | -2.1000 | inactive | [\* 21 \* 25 12 ] | | 4LWT | 10.910 | 2400 | 3900 | -1.6000 | inactive | [\* 11 22 \* 1 ] | | Molecule14 | 10.760 | 3400 | 5500 | -1.6000 | inactive | [13 \* \* 26 16 ] | | Molecule15 | 11.460 | 690 | 18000 | -27 | inactive | [\* 28 25 26 \* ] | | Molecule16 | 10.040 | 18000 | 36000 | -2 | inactive | [13 \* \* 26 16 ] | |

Hypothesis 2

|  |  |  |  |  |  |  |  |  |  |  |  |  |  |  |  |  |  |  |  |  |  |  |  |  |  |  |  |  |  |  |  |  |  |  |  |  |  |  |  |  |  |  |  |  |  |  |  |  |  |  |  |  |  |  |  |  |  |  |  |  |  |  |  |  |  |  |  |  |  |  |  |  |  |  |  |  |  |  |  |  |  |  |  |  |  |  |  |  |  |  |  |  |  |  |  |  |  |  |  |  |  |  |  |  |  |  |  |  |  |  |  |  |  |  |  |  |  |  |  |  |  |  |  |  |  |  |  |  |  |  |  |  |  |  |  |  |  |  |  |  |  |  |  |  |  |  |  |  |  |  |  |  |  |  |  |  |  |  |  |  |  |  |  |  |  |  |  |  |  |  |  |  |  |  |  |  |  |  |  |  |  |  |  |  |  |  |  |  |  |  |  |  |  |  |  |  |  |  |  |  |  |  |  |  |  |  |  |  |  |  |  |  |  |  |  |  |  |  |  |  |  |  |  |  |  |  |  |  |  |  |  |  |  |  |  |  |  |  |
| --- | --- | --- | --- | --- | --- | --- | --- | --- | --- | --- | --- | --- | --- | --- | --- | --- | --- | --- | --- | --- | --- | --- | --- | --- | --- | --- | --- | --- | --- | --- | --- | --- | --- | --- | --- | --- | --- | --- | --- | --- | --- | --- | --- | --- | --- | --- | --- | --- | --- | --- | --- | --- | --- | --- | --- | --- | --- | --- | --- | --- | --- | --- | --- | --- | --- | --- | --- | --- | --- | --- | --- | --- | --- | --- | --- | --- | --- | --- | --- | --- | --- | --- | --- | --- | --- | --- | --- | --- | --- | --- | --- | --- | --- | --- | --- | --- | --- | --- | --- | --- | --- | --- | --- | --- | --- | --- | --- | --- | --- | --- | --- | --- | --- | --- | --- | --- | --- | --- | --- | --- | --- | --- | --- | --- | --- | --- | --- | --- | --- | --- | --- | --- | --- | --- | --- | --- | --- | --- | --- | --- | --- | --- | --- | --- | --- | --- | --- | --- | --- | --- | --- | --- | --- | --- | --- | --- | --- | --- | --- | --- | --- | --- | --- | --- | --- | --- | --- | --- | --- | --- | --- | --- | --- | --- | --- | --- | --- | --- | --- | --- | --- | --- | --- | --- | --- | --- | --- | --- | --- | --- | --- | --- | --- | --- | --- | --- | --- | --- | --- | --- | --- | --- | --- | --- | --- | --- | --- | --- | --- | --- | --- | --- | --- | --- | --- | --- | --- | --- | --- | --- | --- | --- | --- | --- | --- | --- | --- | --- | --- | --- | --- | --- | --- | --- | --- | --- | --- | --- |
| |  |  |  |  |  |  |  |  |  |  |  |  |  |  |  |  |  |  |  | | --- | --- | --- | --- | --- | --- | --- | --- | --- | --- | --- | --- | --- | --- | --- | --- | --- | --- | --- | | |  |  |  |  |  |  |  |  |  |  |  |  |  |  |  |  |  |  | | --- | --- | --- | --- | --- | --- | --- | --- | --- | --- | --- | --- | --- | --- | --- | --- | --- | --- | | |  |  | | --- | --- | | Results | | | Maximum Fit | 17.7925 | | Total Cost | 390.714 | | RMS | 4.36523 | | Correlation | 0.859581 | | |  |  | | --- | --- | | Description | | | Features | Weights | | HBA  HYDROPHOBIC  HYDROPHOBIC  HYDROPHOBIC  RING\_AROMATIC | 3.55850  3.55850  3.55850  3.55850  3.55850 | | | |  | | |  |  |  |  |  |  |  | | --- | --- | --- | --- | --- | --- | --- | | Name | Fit | Est | Act | Err | Status | Mapping | | 4OAS | 13.360 | 3.1000 | 0.1 | 31 | active | [33 \* 28 3 24 ] | | 4QOC\_35T\_C\_201 | 16.390 | 0.0029000 | 0.1 | -34 | active | [30 35 26 3 22 ] | | 5LN2 | 13.890 | 0.91000 | 0.13 | 7 | active | [25 3 40 15 34 ] | | 4erf | 12.950 | 7.9000 | 1.1 | 7.2000 | moderately active | [23 \* 30 3 26 ] | | 4ZTI | 13.640 | 1.6000 | 1.6 | 1 | moderately active | [\* 33 20 31 1 ] | | 4OCC | 13.580 | 1.9000 | 2 | -1.1000 | moderately active | [30 \* 25 3 21 ] | | Molecule1 | 12.080 | 59 | 2 | 29 | moderately active | [23 \* 30 29 1 ] | | Molecule2 | 12.510 | 22 | 2 | 11 | moderately active | [43 45 \* 2 15 ] | | Molecule3 | 12.560 | 20 | 4 | 4.9000 | moderately active | [\* 24 29 31 15 ] | | 4LWV | 13.320 | 3.4000 | 7 | -2.1000 | moderately active | [41 16 26 30 19 ] | | 4OQ3 | 11.790 | 120 | 8 | 15 | moderately active | [\* 20 1 30 2 ] | | Molecule4 | 12.680 | 15 | 16 | -1.1000 | moderately active | [\* 24 29 31 15 ] | | 4DIJ | 11.260 | 390 | 30 | 13 | moderately active | [15 20 3 \* 24 ] | | Molecule5 | 12.940 | 8.2000 | 39 | -4.8000 | moderately active | [26 \* 29 34 15 ] | | 1t4e | 11.850 | 100 | 80 | 1.3000 | moderately active | [\* 18 1 29 2 ] | | Molecule6 | 12.640 | 16 | 81 | -4.9000 | moderately active | [\* 24 29 31 15 ] | | 4HGF | 11.900 | 90 | 90 | 1 | moderately active | [\* 29 38 17 34 ] | | Molecule7 | 12.010 | 69 | 140 | -2 | moderately active | [26 \* 27 34 1 ] | | 1TTV | 11.820 | 110 | 160 | -1.5000 | moderately active | [\* 12 19 24 13 ] | | Molecule8 | 11.380 | 300 | 210 | 1.4000 | moderately active | [\* 26 28 32 15 ] | | Molecule9 | 11.460 | 250 | 230 | 1.1000 | moderately active | [\* 24 26 31 15 ] | | Molecule10 | 11.880 | 93 | 560 | -6 | inactive | [\* 21 27 28 12 ] | | Molecule11 | 11.360 | 310 | 820 | -2.7000 | inactive | [20 21 27 33 12 ] | | Molecule12 | 11.570 | 190 | 1100 | -5.6000 | inactive | [\* 21 27 28 12 ] | | 3JZK | 10.540 | 2100 | 1200 | 1.7000 | inactive | [\* \* 4 22 1 ] | | Molecule13 | 11.140 | 510 | 2100 | -4 | inactive | [\* 21 26 25 12 ] | | 4LWT | 11.120 | 540 | 3900 | -7.2000 | inactive | [27 \* 25 11 1 ] | | Molecule14 | 10.300 | 3600 | 5500 | -1.5000 | inactive | [31 \* 26 25 \* ] | | Molecule15 | 10.580 | 1900 | 18000 | -9.7000 | inactive | [\* \* 26 28 1 ] | | Molecule16 | 10.120 | 5400 | 36000 | -6.7000 | inactive | [\* \* 26 25 1 ] | |

Hypothesis 3

|  |  |  |  |  |  |  |  |  |  |  |  |  |  |  |  |  |  |  |  |  |  |  |  |  |  |  |  |  |  |  |  |  |  |  |  |  |  |  |  |  |  |  |  |  |  |  |  |  |  |  |  |  |  |  |  |  |  |  |  |  |  |  |  |  |  |  |  |  |  |  |  |  |  |  |  |  |  |  |  |  |  |  |  |  |  |  |  |  |  |  |  |  |  |  |  |  |  |  |  |  |  |  |  |  |  |  |  |  |  |  |  |  |  |  |  |  |  |  |  |  |  |  |  |  |  |  |  |  |  |  |  |  |  |  |  |  |  |  |  |  |  |  |  |  |  |  |  |  |  |  |  |  |  |  |  |  |  |  |  |  |  |  |  |  |  |  |  |  |  |  |  |  |  |  |  |  |  |  |  |  |  |  |  |  |  |  |  |  |  |  |  |  |  |  |  |  |  |  |  |  |  |  |  |  |  |  |  |  |  |  |  |  |  |  |  |  |  |  |  |  |  |  |  |  |  |  |  |  |  |  |  |  |  |  |  |  |  |  |
| --- | --- | --- | --- | --- | --- | --- | --- | --- | --- | --- | --- | --- | --- | --- | --- | --- | --- | --- | --- | --- | --- | --- | --- | --- | --- | --- | --- | --- | --- | --- | --- | --- | --- | --- | --- | --- | --- | --- | --- | --- | --- | --- | --- | --- | --- | --- | --- | --- | --- | --- | --- | --- | --- | --- | --- | --- | --- | --- | --- | --- | --- | --- | --- | --- | --- | --- | --- | --- | --- | --- | --- | --- | --- | --- | --- | --- | --- | --- | --- | --- | --- | --- | --- | --- | --- | --- | --- | --- | --- | --- | --- | --- | --- | --- | --- | --- | --- | --- | --- | --- | --- | --- | --- | --- | --- | --- | --- | --- | --- | --- | --- | --- | --- | --- | --- | --- | --- | --- | --- | --- | --- | --- | --- | --- | --- | --- | --- | --- | --- | --- | --- | --- | --- | --- | --- | --- | --- | --- | --- | --- | --- | --- | --- | --- | --- | --- | --- | --- | --- | --- | --- | --- | --- | --- | --- | --- | --- | --- | --- | --- | --- | --- | --- | --- | --- | --- | --- | --- | --- | --- | --- | --- | --- | --- | --- | --- | --- | --- | --- | --- | --- | --- | --- | --- | --- | --- | --- | --- | --- | --- | --- | --- | --- | --- | --- | --- | --- | --- | --- | --- | --- | --- | --- | --- | --- | --- | --- | --- | --- | --- | --- | --- | --- | --- | --- | --- | --- | --- | --- | --- | --- | --- | --- | --- | --- | --- | --- | --- | --- | --- | --- | --- | --- | --- | --- | --- | --- | --- |
| |  |  |  |  |  |  |  |  |  |  |  |  |  |  |  |  |  |  |  | | --- | --- | --- | --- | --- | --- | --- | --- | --- | --- | --- | --- | --- | --- | --- | --- | --- | --- | --- | | |  |  |  |  |  |  |  |  |  |  |  |  |  |  |  |  |  |  | | --- | --- | --- | --- | --- | --- | --- | --- | --- | --- | --- | --- | --- | --- | --- | --- | --- | --- | | |  |  | | --- | --- | | Results | | | Maximum Fit | 13.6274 | | Total Cost | 428.167 | | RMS | 4.71672 | | Correlation | 0.833579 | | |  |  | | --- | --- | | Description | | | Features | Weights | | HBA  HYDROPHOBIC  HYDROPHOBIC  HYDROPHOBIC  HYDROPHOBIC | 2.72549  2.72549  2.72549  2.72549  2.72549 | | | |  | | |  |  |  |  |  |  |  | | --- | --- | --- | --- | --- | --- | --- | | Name | Fit | Est | Act | Err | Status | Mapping | | 4OAS | 10.760 | 1.7000 | 0.1 | 17 | active | [14 4 21 \* 28 ] | | 4QOC\_35T\_C\_201 | 13.590 | 0.0025000 | 0.1 | -40 | active | [13 5 39 35 26 ] | | 5LN2 | 10.180 | 6.4000 | 0.13 | 49 | active | [\* 15 7 6 40 ] | | 4erf | 10.520 | 2.9000 | 1.1 | 2.6000 | moderately active | [14 5 21 \* 30 ] | | 4ZTI | 10.070 | 8.2000 | 1.6 | 5.1000 | moderately active | [\* 20 9 3 18 ] | | 4OCC | 10.210 | 5.9000 | 2 | 3 | moderately active | [13 5 33 \* 25 ] | | Molecule1 | 9.5800 | 25 | 2 | 13 | moderately active | [23 30 29 32 \* ] | | Molecule2 | 10.280 | 5.1000 | 2 | 2.5000 | moderately active | [\* 1 39 45 29 ] | | Molecule3 | 9.9300 | 11 | 4 | 2.8000 | moderately active | [\* 36 21 24 30 ] | | 4LWV | 9.7900 | 16 | 7 | 2.2000 | moderately active | [\* 11 22 30 16 ] | | 4OQ3 | 8.9100 | 120 | 8 | 15 | moderately active | [23 20 8 \* 1 ] | | Molecule4 | 9.9400 | 11 | 16 | -1.4000 | moderately active | [\* 36 21 24 30 ] | | 4DIJ | 8.8300 | 140 | 30 | 4.8000 | moderately active | [31 20 1 3 \* ] | | Molecule5 | 10.270 | 5.1000 | 39 | -7.6000 | moderately active | [\* 27 35 40 21 ] | | 1t4e | 8.1400 | 700 | 80 | 8.7000 | moderately active | [\* 19 3 \* 31 ] | | Molecule6 | 9.9700 | 10 | 81 | -7.8000 | moderately active | [\* 32 21 24 30 ] | | 4HGF | 10.040 | 8.7000 | 90 | -10 | moderately active | [\* 14 28 38 8 ] | | Molecule7 | 8.6500 | 210 | 140 | 1.6000 | moderately active | [25 27 29 33 \* ] | | 1TTV | 8.8000 | 150 | 160 | -1 | moderately active | [\* 9 27 31 15 ] | | Molecule8 | 8.7100 | 190 | 210 | -1.1000 | moderately active | [24 1 32 \* 28 ] | | Molecule9 | 8.1400 | 700 | 230 | 3.1000 | moderately active | [\* 24 26 \* 30 ] | | Molecule10 | 8.1600 | 670 | 560 | 1.2000 | inactive | [\* 21 24 \* 18 ] | | Molecule11 | 8.5500 | 270 | 820 | -3 | inactive | [29 2 33 \* 27 ] | | Molecule12 | 9.1100 | 75 | 1100 | -14 | inactive | [\* 27 24 21 28 ] | | 3JZK | 7.8700 | 1300 | 1200 | 1 | inactive | [\* 22 7 \* 12 ] | | Molecule13 | 8.1400 | 700 | 2100 | -2.9000 | inactive | [\* 26 21 \* 25 ] | | 4LWT | 8.1600 | 660 | 3900 | -5.9000 | inactive | [\* 22 25 \* 17 ] | | Molecule14 | 7.8500 | 1300 | 5500 | -4.1000 | inactive | [14 \* 26 25 \* ] | | Molecule15 | 8.1800 | 640 | 18000 | -28 | inactive | [\* 27 3 25 \* ] | | Molecule16 | 7.2400 | 5500 | 36000 | -6.6000 | inactive | [13 21 26 \* \* ] | |

Hypothesis 4

|  |  |  |  |  |  |  |  |  |  |  |  |  |  |  |  |  |  |  |  |  |  |  |  |  |  |  |  |  |  |  |  |  |  |  |  |  |  |  |  |  |  |  |  |  |  |  |  |  |  |  |  |  |  |  |  |  |  |  |  |  |  |  |  |  |  |  |  |  |  |  |  |  |  |  |  |  |  |  |  |  |  |  |  |  |  |  |  |  |  |  |  |  |  |  |  |  |  |  |  |  |  |  |  |  |  |  |  |  |  |  |  |  |  |  |  |  |  |  |  |  |  |  |  |  |  |  |  |  |  |  |  |  |  |  |  |  |  |  |  |  |  |  |  |  |  |  |  |  |  |  |  |  |  |  |  |  |  |  |  |  |  |  |  |  |  |  |  |  |  |  |  |  |  |  |  |  |  |  |  |  |  |  |  |  |  |  |  |  |  |  |  |  |  |  |  |  |  |  |  |  |  |  |  |  |  |  |  |  |  |  |  |  |  |  |  |  |  |  |  |  |  |  |  |  |  |  |  |  |  |  |  |  |  |  |  |  |  |  |
| --- | --- | --- | --- | --- | --- | --- | --- | --- | --- | --- | --- | --- | --- | --- | --- | --- | --- | --- | --- | --- | --- | --- | --- | --- | --- | --- | --- | --- | --- | --- | --- | --- | --- | --- | --- | --- | --- | --- | --- | --- | --- | --- | --- | --- | --- | --- | --- | --- | --- | --- | --- | --- | --- | --- | --- | --- | --- | --- | --- | --- | --- | --- | --- | --- | --- | --- | --- | --- | --- | --- | --- | --- | --- | --- | --- | --- | --- | --- | --- | --- | --- | --- | --- | --- | --- | --- | --- | --- | --- | --- | --- | --- | --- | --- | --- | --- | --- | --- | --- | --- | --- | --- | --- | --- | --- | --- | --- | --- | --- | --- | --- | --- | --- | --- | --- | --- | --- | --- | --- | --- | --- | --- | --- | --- | --- | --- | --- | --- | --- | --- | --- | --- | --- | --- | --- | --- | --- | --- | --- | --- | --- | --- | --- | --- | --- | --- | --- | --- | --- | --- | --- | --- | --- | --- | --- | --- | --- | --- | --- | --- | --- | --- | --- | --- | --- | --- | --- | --- | --- | --- | --- | --- | --- | --- | --- | --- | --- | --- | --- | --- | --- | --- | --- | --- | --- | --- | --- | --- | --- | --- | --- | --- | --- | --- | --- | --- | --- | --- | --- | --- | --- | --- | --- | --- | --- | --- | --- | --- | --- | --- | --- | --- | --- | --- | --- | --- | --- | --- | --- | --- | --- | --- | --- | --- | --- | --- | --- | --- | --- | --- | --- | --- | --- | --- | --- | --- | --- | --- |
| |  |  |  |  |  |  |  |  |  |  |  |  |  |  |  |  |  |  |  | | --- | --- | --- | --- | --- | --- | --- | --- | --- | --- | --- | --- | --- | --- | --- | --- | --- | --- | --- | | |  |  |  |  |  |  |  |  |  |  |  |  |  |  |  |  |  |  | | --- | --- | --- | --- | --- | --- | --- | --- | --- | --- | --- | --- | --- | --- | --- | --- | --- | --- | | |  |  | | --- | --- | | Results | | | Maximum Fit | 14.4366 | | Total Cost | 435.033 | | RMS | 4.75498 | | Correlation | 0.830607 | | |  |  | | --- | --- | | Description | | | Features | Weights | | HBA  HYDROPHOBIC  HYDROPHOBIC  HYDROPHOBIC  HYDROPHOBIC | 2.88732  2.88732  2.88732  2.88732  2.88732 | | | |  | | |  |  |  |  |  |  |  | | --- | --- | --- | --- | --- | --- | --- | | Name | Fit | Est | Act | Err | Status | Mapping | | 4OAS | 11.220 | 1.3000 | 0.1 | 13 | active | [14 4 21 \* 28 ] | | 4QOC\_35T\_C\_201 | 13.630 | 0.0049000 | 0.1 | -21 | active | [13 5 37 35 26 ] | | 5LN2 | 10.530 | 6.2000 | 0.13 | 48 | active | [\* 15 7 6 40 ] | | 4erf | 11.320 | 1 | 1.1 | -1.1000 | moderately active | [14 4 21 \* 30 ] | | 4ZTI | 10.470 | 7 | 1.6 | 4.4000 | moderately active | [\* 20 9 3 16 ] | | 4OCC | 11.150 | 1.5000 | 2 | -1.3000 | moderately active | [14 5 33 \* 25 ] | | Molecule1 | 9.6900 | 42 | 2 | 21 | moderately active | [23 1 29 \* 32 ] | | Molecule2 | 10.550 | 5.9000 | 2 | 2.9000 | moderately active | [\* 30 21 45 24 ] | | Molecule3 | 10.040 | 19 | 4 | 4.7000 | moderately active | [38 2 31 \* 29 ] | | 4LWV | 10.150 | 15 | 7 | 2.1000 | moderately active | [\* 11 22 33 16 ] | | 4OQ3 | 9.2600 | 120 | 8 | 14 | moderately active | [22 20 8 \* 1 ] | | Molecule4 | 9.3500 | 94 | 16 | 5.9000 | moderately active | [\* 32 21 24 31 ] | | 4DIJ | 8.6300 | 490 | 30 | 16 | moderately active | [\* 28 20 \* 2 ] | | Molecule5 | 10.290 | 11 | 39 | -3.6000 | moderately active | [26 2 34 \* 29 ] | | 1t4e | 8.6000 | 520 | 80 | 6.5000 | moderately active | [\* 19 3 \* 31 ] | | Molecule6 | 9.9300 | 25 | 81 | -3.3000 | moderately active | [\* 1 31 39 29 ] | | 4HGF | 10.570 | 5.7000 | 90 | -16 | moderately active | [6 14 28 \* 39 ] | | Molecule7 | 9.2800 | 110 | 140 | -1.3000 | moderately active | [25 27 34 \* 29 ] | | 1TTV | 9.8700 | 28 | 160 | -5.7000 | moderately active | [\* 19 27 31 10 ] | | Molecule8 | 8.9300 | 250 | 210 | 1.2000 | moderately active | [24 1 33 \* 28 ] | | Molecule9 | 8.6500 | 470 | 230 | 2.1000 | moderately active | [\* 1 30 \* 26 ] | | Molecule10 | 8.6400 | 480 | 560 | -1.2000 | inactive | [\* 2 28 \* 27 ] | | Molecule11 | 9.2300 | 120 | 820 | -6.6000 | inactive | [29 2 33 \* 27 ] | | Molecule12 | 9.2300 | 120 | 1100 | -8.6000 | inactive | [\* 27 24 21 28 ] | | 3JZK | 8.3400 | 950 | 1200 | -1.3000 | inactive | [\* 22 7 \* 12 ] | | Molecule13 | 8.5200 | 640 | 2100 | -3.2000 | inactive | [\* 26 21 \* 25 ] | | 4LWT | 8.6100 | 510 | 3900 | -7.6000 | inactive | [\* 11 22 \* 9 ] | | Molecule14 | 8.1500 | 1500 | 5500 | -3.7000 | inactive | [31 26 25 \* \* ] | | Molecule15 | 8.6000 | 530 | 18000 | -34 | inactive | [\* 32 21 \* 3 ] | | Molecule16 | 7.2700 | 11000 | 36000 | -3.2000 | inactive | [14 26 25 \* \* ] | |

Hypothesis 5

|  |  |  |  |  |  |  |  |  |  |  |  |  |  |  |  |  |  |  |  |  |  |  |  |  |  |  |  |  |  |  |  |  |  |  |  |  |  |  |  |  |  |  |  |  |  |  |  |  |  |  |  |  |  |  |  |  |  |  |  |  |  |  |  |  |  |  |  |  |  |  |  |  |  |  |  |  |  |  |  |  |  |  |  |  |  |  |  |  |  |  |  |  |  |  |  |  |  |  |  |  |  |  |  |  |  |  |  |  |  |  |  |  |  |  |  |  |  |  |  |  |  |  |  |  |  |  |  |  |  |  |  |  |  |  |  |  |  |  |  |  |  |  |  |  |  |  |  |  |  |  |  |  |  |  |  |  |  |  |  |  |  |  |  |  |  |  |  |  |  |  |  |  |  |  |  |  |  |  |  |  |  |  |  |  |  |  |  |  |  |  |  |  |  |  |  |  |  |  |  |  |  |  |  |  |  |  |  |  |  |  |  |  |  |  |  |  |  |  |  |  |  |  |  |  |  |  |  |  |  |  |  |  |  |  |  |  |  |  |
| --- | --- | --- | --- | --- | --- | --- | --- | --- | --- | --- | --- | --- | --- | --- | --- | --- | --- | --- | --- | --- | --- | --- | --- | --- | --- | --- | --- | --- | --- | --- | --- | --- | --- | --- | --- | --- | --- | --- | --- | --- | --- | --- | --- | --- | --- | --- | --- | --- | --- | --- | --- | --- | --- | --- | --- | --- | --- | --- | --- | --- | --- | --- | --- | --- | --- | --- | --- | --- | --- | --- | --- | --- | --- | --- | --- | --- | --- | --- | --- | --- | --- | --- | --- | --- | --- | --- | --- | --- | --- | --- | --- | --- | --- | --- | --- | --- | --- | --- | --- | --- | --- | --- | --- | --- | --- | --- | --- | --- | --- | --- | --- | --- | --- | --- | --- | --- | --- | --- | --- | --- | --- | --- | --- | --- | --- | --- | --- | --- | --- | --- | --- | --- | --- | --- | --- | --- | --- | --- | --- | --- | --- | --- | --- | --- | --- | --- | --- | --- | --- | --- | --- | --- | --- | --- | --- | --- | --- | --- | --- | --- | --- | --- | --- | --- | --- | --- | --- | --- | --- | --- | --- | --- | --- | --- | --- | --- | --- | --- | --- | --- | --- | --- | --- | --- | --- | --- | --- | --- | --- | --- | --- | --- | --- | --- | --- | --- | --- | --- | --- | --- | --- | --- | --- | --- | --- | --- | --- | --- | --- | --- | --- | --- | --- | --- | --- | --- | --- | --- | --- | --- | --- | --- | --- | --- | --- | --- | --- | --- | --- | --- | --- | --- | --- | --- | --- | --- | --- | --- |
| |  |  |  |  |  |  |  |  |  |  |  |  |  |  |  |  |  |  |  | | --- | --- | --- | --- | --- | --- | --- | --- | --- | --- | --- | --- | --- | --- | --- | --- | --- | --- | --- | | |  |  |  |  |  |  |  |  |  |  |  |  |  |  |  |  |  |  | | --- | --- | --- | --- | --- | --- | --- | --- | --- | --- | --- | --- | --- | --- | --- | --- | --- | --- | | |  |  | | --- | --- | | Results | | | Maximum Fit | 17.2322 | | Total Cost | 436.479 | | RMS | 4.71479 | | Correlation | 0.833846 | | |  |  | | --- | --- | | Description | | | Features | Weights | | HYDROPHOBIC  HYDROPHOBIC  HYDROPHOBIC  HYDROPHOBIC  HYDROPHOBIC | 3.44645  3.44645  3.44645  3.44645  3.44645 | | | |  | | |  |  |  |  |  |  |  | | --- | --- | --- | --- | --- | --- | --- | | Name | Fit | Est | Act | Err | Status | Mapping | | 4OAS | 13.450 | 12 | 0.1 | 120 | active | [\* 5 28 21 3 ] | | 4QOC\_35T\_C\_201 | 15 | 0.33000 | 0.1 | 3.3000 | active | [34 5 26 39 3 ] | | 5LN2 | 15.040 | 0.30000 | 0.13 | 2.3000 | active | [6 19 40 7 15 ] | | 4erf | 13.410 | 13 | 1.1 | 12 | moderately active | [\* 5 30 21 3 ] | | 4ZTI | 13.520 | 9.8000 | 1.6 | 6.1000 | moderately active | [16 3 \* 20 31 ] | | 4OCC | 13.330 | 15 | 2 | 7.7000 | moderately active | [\* 5 25 33 3 ] | | Molecule1 | 13.510 | 10 | 2 | 5 | moderately active | [\* 2 32 29 30 ] | | Molecule2 | 14.720 | 0.63000 | 2 | -3.2000 | moderately active | [45 2 29 39 24 ] | | Molecule3 | 13.110 | 26 | 4 | 6.4000 | moderately active | [\* 1 29 31 24 ] | | 4LWV | 13.500 | 10 | 7 | 1.5000 | moderately active | [\* 33 11 4 30 ] | | 4OQ3 | 12.990 | 33 | 8 | 4.2000 | moderately active | [\* 18 1 27 20 ] | | Molecule4 | 13.230 | 19 | 16 | 1.2000 | moderately active | [\* 2 29 30 24 ] | | 4DIJ | 12.090 | 270 | 30 | 8.8000 | moderately active | [\* 27 2 20 3 ] | | Molecule5 | 13.370 | 14 | 39 | -2.8000 | moderately active | [40 2 29 \* 27 ] | | 1t4e | 12.430 | 120 | 80 | 1.5000 | moderately active | [\* 19 29 3 18 ] | | Molecule6 | 13.220 | 20 | 81 | -4.1000 | moderately active | [\* 2 29 31 24 ] | | 4HGF | 13.380 | 14 | 90 | -6.6000 | moderately active | [38 \* 23 17 8 ] | | Molecule7 | 13.550 | 9.3000 | 140 | -15 | moderately active | [\* 2 29 34 27 ] | | 1TTV | 13.350 | 15 | 160 | -11 | moderately active | [31 9 \* 27 14 ] | | Molecule8 | 13.240 | 19 | 210 | -11 | moderately active | [\* 2 28 32 26 ] | | Molecule9 | 11.660 | 720 | 230 | 3.1000 | moderately active | [26 24 \* 31 2 ] | | Molecule10 | 13.130 | 24 | 560 | -23 | inactive | [\* 1 27 28 21 ] | | Molecule11 | 12.630 | 77 | 820 | -11 | inactive | [\* 2 27 33 21 ] | | Molecule12 | 12.600 | 82 | 1100 | -13 | inactive | [\* 2 27 28 21 ] | | 3JZK | 10.960 | 3600 | 1200 | 3 | inactive | [\* 19 7 4 22 ] | | Molecule13 | 11.200 | 2100 | 2100 | -1 | inactive | [\* 2 26 25 21 ] | | 4LWT | 11.910 | 410 | 3900 | -9.6000 | inactive | [\* 17 22 8 11 ] | | Molecule14 | 10.310 | 16000 | 5500 | 2.9000 | inactive | [\* 3 25 \* 26 ] | | Molecule15 | 10.410 | 13000 | 18000 | -1.4000 | inactive | [28 3 25 \* 26 ] | | Molecule16 | 10.280 | 17000 | 36000 | -2.1000 | inactive | [\* 3 21 \* 26 ] | |

Hypothesis 6

|  |  |  |  |  |  |  |  |  |  |  |  |  |  |  |  |  |  |  |  |  |  |  |  |  |  |  |  |  |  |  |  |  |  |  |  |  |  |  |  |  |  |  |  |  |  |  |  |  |  |  |  |  |  |  |  |  |  |  |  |  |  |  |  |  |  |  |  |  |  |  |  |  |  |  |  |  |  |  |  |  |  |  |  |  |  |  |  |  |  |  |  |  |  |  |  |  |  |  |  |  |  |  |  |  |  |  |  |  |  |  |  |  |  |  |  |  |  |  |  |  |  |  |  |  |  |  |  |  |  |  |  |  |  |  |  |  |  |  |  |  |  |  |  |  |  |  |  |  |  |  |  |  |  |  |  |  |  |  |  |  |  |  |  |  |  |  |  |  |  |  |  |  |  |  |  |  |  |  |  |  |  |  |  |  |  |  |  |  |  |  |  |  |  |  |  |  |  |  |  |  |  |  |  |  |  |  |  |  |  |  |  |  |  |  |  |  |  |  |  |  |  |  |  |  |  |  |  |  |  |  |  |  |  |  |  |  |  |  |
| --- | --- | --- | --- | --- | --- | --- | --- | --- | --- | --- | --- | --- | --- | --- | --- | --- | --- | --- | --- | --- | --- | --- | --- | --- | --- | --- | --- | --- | --- | --- | --- | --- | --- | --- | --- | --- | --- | --- | --- | --- | --- | --- | --- | --- | --- | --- | --- | --- | --- | --- | --- | --- | --- | --- | --- | --- | --- | --- | --- | --- | --- | --- | --- | --- | --- | --- | --- | --- | --- | --- | --- | --- | --- | --- | --- | --- | --- | --- | --- | --- | --- | --- | --- | --- | --- | --- | --- | --- | --- | --- | --- | --- | --- | --- | --- | --- | --- | --- | --- | --- | --- | --- | --- | --- | --- | --- | --- | --- | --- | --- | --- | --- | --- | --- | --- | --- | --- | --- | --- | --- | --- | --- | --- | --- | --- | --- | --- | --- | --- | --- | --- | --- | --- | --- | --- | --- | --- | --- | --- | --- | --- | --- | --- | --- | --- | --- | --- | --- | --- | --- | --- | --- | --- | --- | --- | --- | --- | --- | --- | --- | --- | --- | --- | --- | --- | --- | --- | --- | --- | --- | --- | --- | --- | --- | --- | --- | --- | --- | --- | --- | --- | --- | --- | --- | --- | --- | --- | --- | --- | --- | --- | --- | --- | --- | --- | --- | --- | --- | --- | --- | --- | --- | --- | --- | --- | --- | --- | --- | --- | --- | --- | --- | --- | --- | --- | --- | --- | --- | --- | --- | --- | --- | --- | --- | --- | --- | --- | --- | --- | --- | --- | --- | --- | --- | --- | --- | --- | --- |
| |  |  |  |  |  |  |  |  |  |  |  |  |  |  |  |  |  |  |  | | --- | --- | --- | --- | --- | --- | --- | --- | --- | --- | --- | --- | --- | --- | --- | --- | --- | --- | --- | | |  |  |  |  |  |  |  |  |  |  |  |  |  |  |  |  |  |  | | --- | --- | --- | --- | --- | --- | --- | --- | --- | --- | --- | --- | --- | --- | --- | --- | --- | --- | | |  |  | | --- | --- | | Results | | | Maximum Fit | 16.5457 | | Total Cost | 439.345 | | RMS | 4.7496 | | Correlation | 0.831099 | | |  |  | | --- | --- | | Description | | | Features | Weights | | HBA  HYDROPHOBIC  HYDROPHOBIC  HYDROPHOBIC  RING\_AROMATIC | 3.30915  3.30915  3.30915  3.30915  3.30915 | | | |  | | |  |  |  |  |  |  |  | | --- | --- | --- | --- | --- | --- | --- | | Name | Fit | Est | Act | Err | Status | Mapping | | 4OAS | 12.100 | 3.2000 | 0.1 | 32 | active | [13 \* 3 21 24 ] | | 4QOC\_35T\_C\_201 | 14.530 | 0.012000 | 0.1 | -8.5000 | active | [13 34 3 39 22 ] | | 5LN2 | 12.550 | 1.1000 | 0.13 | 8.7000 | active | [\* 6 15 7 34 ] | | 4erf | 11.940 | 4.5000 | 1.1 | 4.1000 | moderately active | [13 \* 3 21 26 ] | | 4ZTI | 11.390 | 16 | 1.6 | 10 | moderately active | [\* 40 3 16 1 ] | | 4OCC | 11.500 | 12 | 2 | 6.2000 | moderately active | [13 \* 33 5 21 ] | | Molecule1 | 11.380 | 16 | 2 | 8.2000 | moderately active | [\* 25 30 28 12 ] | | Molecule2 | 12.640 | 0.91000 | 2 | -2.2000 | moderately active | [\* 45 24 39 15 ] | | Molecule3 | 10.800 | 63 | 4 | 16 | moderately active | [38 \* 24 30 15 ] | | 4LWV | 12.430 | 1.5000 | 7 | -4.7000 | moderately active | [\* 11 30 4 19 ] | | 4OQ3 | 9.7600 | 700 | 8 | 87 | moderately active | [\* 18 30 1 \* ] | | Molecule4 | 10.940 | 46 | 16 | 2.9000 | moderately active | [\* 32 24 31 15 ] | | 4DIJ | 10.680 | 83 | 30 | 2.8000 | moderately active | [38 27 20 \* 5 ] | | Molecule5 | 12.150 | 2.8000 | 39 | -14 | moderately active | [\* 40 27 34 15 ] | | 1t4e | 11.310 | 19 | 80 | -4.1000 | moderately active | [11 \* 1 19 26 ] | | Molecule6 | 10.330 | 180 | 81 | 2.3000 | moderately active | [38 30 1 \* 15 ] | | 4HGF | 10.560 | 110 | 90 | 1.2000 | moderately active | [\* 14 38 17 25 ] | | Molecule7 | 11.030 | 37 | 140 | -3.7000 | moderately active | [26 \* 27 33 15 ] | | 1TTV | 11.460 | 14 | 160 | -12 | moderately active | [\* 24 19 27 6 ] | | Molecule8 | 10.330 | 190 | 210 | -1.1000 | moderately active | [\* 12 26 32 15 ] | | Molecule9 | 9.8000 | 630 | 230 | 2.8000 | moderately active | [\* \* 24 31 15 ] | | Molecule10 | 10.010 | 390 | 560 | -1.4000 | inactive | [\* 24 21 29 12 ] | | Molecule11 | 10.350 | 180 | 820 | -4.6000 | inactive | [30 \* 21 35 12 ] | | Molecule12 | 10.190 | 260 | 1100 | -4.2000 | inactive | [\* 24 21 29 12 ] | | 3JZK | 9.7700 | 670 | 1200 | -1.8000 | inactive | [\* 4 22 7 \* ] | | Molecule13 | 9.7700 | 670 | 2100 | -3.1000 | inactive | [\* 21 25 \* 12 ] | | 4LWT | 9.4100 | 1500 | 3900 | -2.5000 | inactive | [\* \* 11 22 1 ] | | Molecule14 | 9.7200 | 760 | 5500 | -7.2000 | inactive | [31 \* \* 21 1 ] | | Molecule15 | 9.8500 | 560 | 18000 | -33 | inactive | [\* 26 32 21 \* ] | | Molecule16 | 8.3600 | 17000 | 36000 | -2.1000 | inactive | [14 \* \* 26 16 ] | |

Hypothesis 7

|  |  |  |  |  |  |  |  |  |  |  |  |  |  |  |  |  |  |  |  |  |  |  |  |  |  |  |  |  |  |  |  |  |  |  |  |  |  |  |  |  |  |  |  |  |  |  |  |  |  |  |  |  |  |  |  |  |  |  |  |  |  |  |  |  |  |  |  |  |  |  |  |  |  |  |  |  |  |  |  |  |  |  |  |  |  |  |  |  |  |  |  |  |  |  |  |  |  |  |  |  |  |  |  |  |  |  |  |  |  |  |  |  |  |  |  |  |  |  |  |  |  |  |  |  |  |  |  |  |  |  |  |  |  |  |  |  |  |  |  |  |  |  |  |  |  |  |  |  |  |  |  |  |  |  |  |  |  |  |  |  |  |  |  |  |  |  |  |  |  |  |  |  |  |  |  |  |  |  |  |  |  |  |  |  |  |  |  |  |  |  |  |  |  |  |  |  |  |  |  |  |  |  |  |  |  |  |  |  |  |  |  |  |  |  |  |  |  |  |  |  |  |  |  |  |  |  |  |  |  |  |  |  |  |  |  |  |  |  |
| --- | --- | --- | --- | --- | --- | --- | --- | --- | --- | --- | --- | --- | --- | --- | --- | --- | --- | --- | --- | --- | --- | --- | --- | --- | --- | --- | --- | --- | --- | --- | --- | --- | --- | --- | --- | --- | --- | --- | --- | --- | --- | --- | --- | --- | --- | --- | --- | --- | --- | --- | --- | --- | --- | --- | --- | --- | --- | --- | --- | --- | --- | --- | --- | --- | --- | --- | --- | --- | --- | --- | --- | --- | --- | --- | --- | --- | --- | --- | --- | --- | --- | --- | --- | --- | --- | --- | --- | --- | --- | --- | --- | --- | --- | --- | --- | --- | --- | --- | --- | --- | --- | --- | --- | --- | --- | --- | --- | --- | --- | --- | --- | --- | --- | --- | --- | --- | --- | --- | --- | --- | --- | --- | --- | --- | --- | --- | --- | --- | --- | --- | --- | --- | --- | --- | --- | --- | --- | --- | --- | --- | --- | --- | --- | --- | --- | --- | --- | --- | --- | --- | --- | --- | --- | --- | --- | --- | --- | --- | --- | --- | --- | --- | --- | --- | --- | --- | --- | --- | --- | --- | --- | --- | --- | --- | --- | --- | --- | --- | --- | --- | --- | --- | --- | --- | --- | --- | --- | --- | --- | --- | --- | --- | --- | --- | --- | --- | --- | --- | --- | --- | --- | --- | --- | --- | --- | --- | --- | --- | --- | --- | --- | --- | --- | --- | --- | --- | --- | --- | --- | --- | --- | --- | --- | --- | --- | --- | --- | --- | --- | --- | --- | --- | --- | --- | --- | --- | --- | --- |
| |  |  |  |  |  |  |  |  |  |  |  |  |  |  |  |  |  |  |  | | --- | --- | --- | --- | --- | --- | --- | --- | --- | --- | --- | --- | --- | --- | --- | --- | --- | --- | --- | | |  |  |  |  |  |  |  |  |  |  |  |  |  |  |  |  |  |  | | --- | --- | --- | --- | --- | --- | --- | --- | --- | --- | --- | --- | --- | --- | --- | --- | --- | --- | | |  |  | | --- | --- | | Results | | | Maximum Fit | 16.5052 | | Total Cost | 448.054 | | RMS | 4.81113 | | Correlation | 0.826229 | | |  |  | | --- | --- | | Description | | | Features | Weights | | HBA  HYDROPHOBIC  HYDROPHOBIC  HYDROPHOBIC  RING\_AROMATIC | 3.30104  3.30104  3.30104  3.30104  3.30104 | | | |  | | |  |  |  |  |  |  |  | | --- | --- | --- | --- | --- | --- | --- | | Name | Fit | Est | Act | Err | Status | Mapping | | 4OAS | 12.450 | 2.5000 | 0.1 | 25 | active | [13 21 3 5 24 ] | | 4QOC\_35T\_C\_201 | 14.360 | 0.031000 | 0.1 | -3.2000 | active | [14 3 34 39 22 ] | | 5LN2 | 12.520 | 2.2000 | 0.13 | 17 | active | [\* 15 6 7 34 ] | | 4erf | 12.320 | 3.4000 | 1.1 | 3.1000 | moderately active | [13 3 \* 21 26 ] | | 4ZTI | 11.360 | 31 | 1.6 | 19 | moderately active | [\* 3 40 16 1 ] | | 4OCC | 11.850 | 10 | 2 | 5 | moderately active | [13 3 25 33 \* ] | | Molecule1 | 12.120 | 5.4000 | 2 | 2.7000 | moderately active | [23 30 \* 29 12 ] | | Molecule2 | 12.610 | 1.7000 | 2 | -1.2000 | moderately active | [\* 24 45 39 15 ] | | Molecule3 | 11.750 | 13 | 4 | 3.2000 | moderately active | [38 24 31 \* 15 ] | | 4LWV | 12.400 | 2.8000 | 7 | -2.5000 | moderately active | [\* 30 11 4 19 ] | | 4OQ3 | 11.490 | 23 | 8 | 2.9000 | moderately active | [22 20 \* 28 2 ] | | Molecule4 | 10.910 | 88 | 16 | 5.5000 | moderately active | [\* 24 32 31 15 ] | | 4DIJ | 10.150 | 500 | 30 | 17 | moderately active | [38 3 \* 20 5 ] | | Molecule5 | 12.120 | 5.3000 | 39 | -7.3000 | moderately active | [\* 27 40 34 15 ] | | 1t4e | 11.020 | 68 | 80 | -1.2000 | moderately active | [11 1 \* 19 26 ] | | Molecule6 | 10.110 | 560 | 81 | 6.9000 | moderately active | [23 24 39 36 \* ] | | 4HGF | 10.530 | 210 | 90 | 2.3000 | moderately active | [\* 38 14 17 25 ] | | Molecule7 | 11.510 | 22 | 140 | -6.2000 | moderately active | [26 27 \* 34 15 ] | | 1TTV | 11.430 | 26 | 160 | -6.1000 | moderately active | [\* 19 24 27 6 ] | | Molecule8 | 11.990 | 7.3000 | 210 | -29 | moderately active | [24 26 \* 33 15 ] | | Molecule9 | 9.7700 | 1200 | 230 | 5.2000 | moderately active | [\* 24 \* 31 15 ] | | Molecule10 | 9.9900 | 730 | 560 | 1.3000 | inactive | [\* 21 24 29 12 ] | | Molecule11 | 12.020 | 6.8000 | 820 | -120 | inactive | [30 1 \* 33 12 ] | | Molecule12 | 10.170 | 480 | 1100 | -2.2000 | inactive | [\* 21 24 29 12 ] | | 3JZK | 9.7500 | 1300 | 1200 | 1 | inactive | [\* 22 4 7 \* ] | | Molecule13 | 9.7500 | 1300 | 2100 | -1.6000 | inactive | [\* 25 21 \* 12 ] | | 4LWT | 9.6300 | 1600 | 3900 | -2.4000 | inactive | [27 \* 11 9 \* ] | | Molecule14 | 9.6200 | 1700 | 5500 | -3.2000 | inactive | [29 \* 25 \* 1 ] | | Molecule15 | 9.8300 | 1100 | 18000 | -17 | inactive | [\* 32 26 21 \* ] | | Molecule16 | 9.0900 | 5700 | 36000 | -6.4000 | inactive | [14 26 \* 25 \* ] | |

Hypothesis 8

|  |  |  |  |  |  |  |  |  |  |  |  |  |  |  |  |  |  |  |  |  |  |  |  |  |  |  |  |  |  |  |  |  |  |  |  |  |  |  |  |  |  |  |  |  |  |  |  |  |  |  |  |  |  |  |  |  |  |  |  |  |  |  |  |  |  |  |  |  |  |  |  |  |  |  |  |  |  |  |  |  |  |  |  |  |  |  |  |  |  |  |  |  |  |  |  |  |  |  |  |  |  |  |  |  |  |  |  |  |  |  |  |  |  |  |  |  |  |  |  |  |  |  |  |  |  |  |  |  |  |  |  |  |  |  |  |  |  |  |  |  |  |  |  |  |  |  |  |  |  |  |  |  |  |  |  |  |  |  |  |  |  |  |  |  |  |  |  |  |  |  |  |  |  |  |  |  |  |  |  |  |  |  |  |  |  |  |  |  |  |  |  |  |  |  |  |  |  |  |  |  |  |  |  |  |  |  |  |  |  |  |  |  |  |  |  |  |  |  |  |  |  |  |  |  |  |  |  |  |  |  |  |  |  |  |  |  |  |  |
| --- | --- | --- | --- | --- | --- | --- | --- | --- | --- | --- | --- | --- | --- | --- | --- | --- | --- | --- | --- | --- | --- | --- | --- | --- | --- | --- | --- | --- | --- | --- | --- | --- | --- | --- | --- | --- | --- | --- | --- | --- | --- | --- | --- | --- | --- | --- | --- | --- | --- | --- | --- | --- | --- | --- | --- | --- | --- | --- | --- | --- | --- | --- | --- | --- | --- | --- | --- | --- | --- | --- | --- | --- | --- | --- | --- | --- | --- | --- | --- | --- | --- | --- | --- | --- | --- | --- | --- | --- | --- | --- | --- | --- | --- | --- | --- | --- | --- | --- | --- | --- | --- | --- | --- | --- | --- | --- | --- | --- | --- | --- | --- | --- | --- | --- | --- | --- | --- | --- | --- | --- | --- | --- | --- | --- | --- | --- | --- | --- | --- | --- | --- | --- | --- | --- | --- | --- | --- | --- | --- | --- | --- | --- | --- | --- | --- | --- | --- | --- | --- | --- | --- | --- | --- | --- | --- | --- | --- | --- | --- | --- | --- | --- | --- | --- | --- | --- | --- | --- | --- | --- | --- | --- | --- | --- | --- | --- | --- | --- | --- | --- | --- | --- | --- | --- | --- | --- | --- | --- | --- | --- | --- | --- | --- | --- | --- | --- | --- | --- | --- | --- | --- | --- | --- | --- | --- | --- | --- | --- | --- | --- | --- | --- | --- | --- | --- | --- | --- | --- | --- | --- | --- | --- | --- | --- | --- | --- | --- | --- | --- | --- | --- | --- | --- | --- | --- | --- | --- | --- |
| |  |  |  |  |  |  |  |  |  |  |  |  |  |  |  |  |  |  |  | | --- | --- | --- | --- | --- | --- | --- | --- | --- | --- | --- | --- | --- | --- | --- | --- | --- | --- | --- | | |  |  |  |  |  |  |  |  |  |  |  |  |  |  |  |  |  |  | | --- | --- | --- | --- | --- | --- | --- | --- | --- | --- | --- | --- | --- | --- | --- | --- | --- | --- | | |  |  | | --- | --- | | Results | | | Maximum Fit | 13.1625 | | Total Cost | 460.114 | | RMS | 4.94201 | | Correlation | 0.815472 | | |  |  | | --- | --- | | Description | | | Features | Weights | | HBA  HYDROPHOBIC  HYDROPHOBIC  HYDROPHOBIC  HYDROPHOBIC | 2.63249  2.63249  2.63249  2.63249  2.63249 | | | |  | | |  |  |  |  |  |  |  | | --- | --- | --- | --- | --- | --- | --- | | Name | Fit | Est | Act | Err | Status | Mapping | | 4OAS | 12.620 | 0.20000 | 0.1 | 2 | active | [14 4 21 28 3 ] | | 4QOC\_35T\_C\_201 | 13.160 | 0.056000 | 0.1 | -1.8000 | active | [14 5 39 26 3 ] | | 5LN2 | 10.140 | 60 | 0.13 | 460 | active | [\* 19 7 40 15 ] | | 4erf | 13 | 0.081000 | 1.1 | -14 | moderately active | [13 5 21 30 3 ] | | 4ZTI | 11.060 | 7.1000 | 1.6 | 4.4000 | moderately active | [30 1 11 31 20 ] | | 4OCC | 11.040 | 7.5000 | 2 | 3.8000 | moderately active | [14 5 33 25 3 ] | | Molecule1 | 10.450 | 29 | 2 | 14 | moderately active | [\* 2 28 32 30 ] | | Molecule2 | 10.690 | 17 | 2 | 8.3000 | moderately active | [37 6 45 39 24 ] | | Molecule3 | 10.460 | 28 | 4 | 7.1000 | moderately active | [\* 1 31 29 24 ] | | 4LWV | 11.090 | 6.7000 | 7 | -1 | moderately active | [32 22 11 16 26 ] | | 4OQ3 | 10.320 | 39 | 8 | 4.9000 | moderately active | [\* 18 27 1 20 ] | | Molecule4 | 10.450 | 29 | 16 | 1.8000 | moderately active | [\* 2 31 29 24 ] | | 4DIJ | 10.030 | 76 | 30 | 2.5000 | moderately active | [\* 27 20 2 3 ] | | Molecule5 | 10.490 | 26 | 39 | -1.5000 | moderately active | [26 6 21 34 27 ] | | 1t4e | 10.090 | 66 | 80 | -1.2000 | moderately active | [\* 19 3 31 18 ] | | Molecule6 | 10.510 | 25 | 81 | -3.2000 | moderately active | [\* 2 31 29 24 ] | | 4HGF | 10.240 | 47 | 90 | -1.9000 | moderately active | [6 37 17 14 38 ] | | Molecule7 | 9.8900 | 110 | 140 | -1.3000 | moderately active | [\* 1 34 12 27 ] | | 1TTV | 9.5100 | 250 | 160 | 1.6000 | moderately active | [\* 10 27 24 12 ] | | Molecule8 | 10.430 | 31 | 210 | -6.9000 | moderately active | [\* 2 32 28 26 ] | | Molecule9 | 8.7300 | 1500 | 230 | 6.6000 | moderately active | [\* 1 31 20 24 ] | | Molecule10 | 9.6200 | 200 | 560 | -2.9000 | inactive | [\* 2 28 24 21 ] | | Molecule11 | 10.300 | 41 | 820 | -20 | inactive | [\* 2 33 27 21 ] | | Molecule12 | 9.3300 | 380 | 1100 | -2.8000 | inactive | [\* 1 28 24 21 ] | | 3JZK | 9.1800 | 540 | 1200 | -2.3000 | inactive | [\* 19 4 7 22 ] | | Molecule13 | 9.5600 | 230 | 2100 | -9.2000 | inactive | [\* 2 25 26 21 ] | | 4LWT | 8.4000 | 3300 | 3900 | -1.2000 | inactive | [\* 17 22 25 11 ] | | Molecule14 | 7.9800 | 8600 | 5500 | 1.6000 | inactive | [24 2 25 \* 26 ] | | Molecule15 | 9.7900 | 130 | 18000 | -140 | inactive | [\* 21 26 27 25 ] | | Molecule16 | 7.8600 | 11000 | 36000 | -3.3000 | inactive | [\* 21 26 \* 25 ] | |

Hypothesis 9

|  |  |  |  |  |  |  |  |  |  |  |  |  |  |  |  |  |  |  |  |  |  |  |  |  |  |  |  |  |  |  |  |  |  |  |  |  |  |  |  |  |  |  |  |  |  |  |  |  |  |  |  |  |  |  |  |  |  |  |  |  |  |  |  |  |  |  |  |  |  |  |  |  |  |  |  |  |  |  |  |  |  |  |  |  |  |  |  |  |  |  |  |  |  |  |  |  |  |  |  |  |  |  |  |  |  |  |  |  |  |  |  |  |  |  |  |  |  |  |  |  |  |  |  |  |  |  |  |  |  |  |  |  |  |  |  |  |  |  |  |  |  |  |  |  |  |  |  |  |  |  |  |  |  |  |  |  |  |  |  |  |  |  |  |  |  |  |  |  |  |  |  |  |  |  |  |  |  |  |  |  |  |  |  |  |  |  |  |  |  |  |  |  |  |  |  |  |  |  |  |  |  |  |  |  |  |  |  |  |  |  |  |  |  |  |  |  |  |  |  |  |  |  |  |  |  |  |  |  |  |  |  |  |  |  |  |  |  |  |
| --- | --- | --- | --- | --- | --- | --- | --- | --- | --- | --- | --- | --- | --- | --- | --- | --- | --- | --- | --- | --- | --- | --- | --- | --- | --- | --- | --- | --- | --- | --- | --- | --- | --- | --- | --- | --- | --- | --- | --- | --- | --- | --- | --- | --- | --- | --- | --- | --- | --- | --- | --- | --- | --- | --- | --- | --- | --- | --- | --- | --- | --- | --- | --- | --- | --- | --- | --- | --- | --- | --- | --- | --- | --- | --- | --- | --- | --- | --- | --- | --- | --- | --- | --- | --- | --- | --- | --- | --- | --- | --- | --- | --- | --- | --- | --- | --- | --- | --- | --- | --- | --- | --- | --- | --- | --- | --- | --- | --- | --- | --- | --- | --- | --- | --- | --- | --- | --- | --- | --- | --- | --- | --- | --- | --- | --- | --- | --- | --- | --- | --- | --- | --- | --- | --- | --- | --- | --- | --- | --- | --- | --- | --- | --- | --- | --- | --- | --- | --- | --- | --- | --- | --- | --- | --- | --- | --- | --- | --- | --- | --- | --- | --- | --- | --- | --- | --- | --- | --- | --- | --- | --- | --- | --- | --- | --- | --- | --- | --- | --- | --- | --- | --- | --- | --- | --- | --- | --- | --- | --- | --- | --- | --- | --- | --- | --- | --- | --- | --- | --- | --- | --- | --- | --- | --- | --- | --- | --- | --- | --- | --- | --- | --- | --- | --- | --- | --- | --- | --- | --- | --- | --- | --- | --- | --- | --- | --- | --- | --- | --- | --- | --- | --- | --- | --- | --- | --- | --- | --- |
| |  |  |  |  |  |  |  |  |  |  |  |  |  |  |  |  |  |  |  | | --- | --- | --- | --- | --- | --- | --- | --- | --- | --- | --- | --- | --- | --- | --- | --- | --- | --- | --- | | |  |  |  |  |  |  |  |  |  |  |  |  |  |  |  |  |  |  | | --- | --- | --- | --- | --- | --- | --- | --- | --- | --- | --- | --- | --- | --- | --- | --- | --- | --- | | |  |  | | --- | --- | | Results | | | Maximum Fit | 16.4833 | | Total Cost | 464.998 | | RMS | 4.82196 | | Correlation | 0.825712 | | |  |  | | --- | --- | | Description | | | Features | Weights | | HBA  HYDROPHOBIC  HYDROPHOBIC  HYDROPHOBIC | 4.12084  4.12084  4.12084  4.12084 | | | |  | | |  |  |  |  |  |  |  | | --- | --- | --- | --- | --- | --- | --- | | Name | Fit | Est | Act | Err | Status | Mapping | | 4OAS | 14.610 | 1.7000 | 0.1 | 17 | active | [14 26 5 21 ] | | 4QOC\_35T\_C\_201 | 14.690 | 1.5000 | 0.1 | 15 | active | [14 39 25 35 ] | | 5LN2 | 15.300 | 0.36000 | 0.13 | 2.7000 | active | [28 6 3 36 ] | | 4erf | 14.700 | 1.4000 | 1.1 | 1.3000 | moderately active | [14 29 3 21 ] | | 4ZTI | 14.580 | 1.9000 | 1.6 | 1.2000 | moderately active | [30 33 40 1 ] | | 4OCC | 14.900 | 0.89000 | 2 | -2.3000 | moderately active | [14 24 33 3 ] | | Molecule1 | 13.380 | 30 | 2 | 15 | moderately active | [23 18 29 2 ] | | Molecule2 | 12.890 | 92 | 2 | 46 | moderately active | [23 39 45 21 ] | | Molecule3 | 14.060 | 6.1000 | 4 | 1.5000 | moderately active | [23 24 32 29 ] | | 4LWV | 15.090 | 0.58000 | 7 | -12 | moderately active | [32 4 11 26 ] | | 4OQ3 | 15.090 | 0.58000 | 8 | -14 | moderately active | [23 7 30 20 ] | | Molecule4 | 12.300 | 360 | 16 | 22 | moderately active | [\* 31 21 32 ] | | 4DIJ | 13.050 | 63 | 30 | 2.1000 | moderately active | [31 1 3 20 ] | | Molecule5 | 14.250 | 4 | 39 | -9.7000 | moderately active | [26 40 34 27 ] | | 1t4e | 12.060 | 620 | 80 | 7.8000 | moderately active | [\* 31 2 18 ] | | Molecule6 | 12.290 | 360 | 81 | 4.5000 | moderately active | [\* 21 30 24 ] | | 4HGF | 12.260 | 390 | 90 | 4.4000 | moderately active | [\* 14 17 36 ] | | Molecule7 | 13.550 | 20 | 140 | -6.9000 | moderately active | [25 21 33 27 ] | | 1TTV | 12.310 | 350 | 160 | 2.2000 | moderately active | [\* 9 16 27 ] | | Molecule8 | 12.290 | 370 | 210 | 1.7000 | moderately active | [\* 33 12 28 ] | | Molecule9 | 12.190 | 460 | 230 | 2 | moderately active | [\* 21 31 24 ] | | Molecule10 | 12.350 | 320 | 560 | -1.8000 | inactive | [\* 28 24 27 ] | | Molecule11 | 12.320 | 340 | 820 | -2.4000 | inactive | [\* 18 2 33 ] | | Molecule12 | 12.360 | 310 | 1100 | -3.4000 | inactive | [\* 28 24 27 ] | | 3JZK | 12.060 | 620 | 1200 | -2 | inactive | [\* 29 4 22 ] | | Molecule13 | 11.940 | 820 | 2100 | -2.5000 | inactive | [\* 17 1 25 ] | | 4LWT | 12.160 | 490 | 3900 | -7.9000 | inactive | [\* 8 22 11 ] | | Molecule14 | 11.910 | 870 | 5500 | -6.3000 | inactive | [31 3 21 \* ] | | Molecule15 | 12.250 | 400 | 18000 | -46 | inactive | [\* 22 27 26 ] | | Molecule16 | 11.440 | 2600 | 36000 | -14 | inactive | [14 26 \* 21 ] | |

Hypothesis 10

|  |  |  |  |  |  |  |  |  |  |  |  |  |  |  |  |  |  |  |  |  |  |  |  |  |  |  |  |  |  |  |  |  |  |  |  |  |  |  |  |  |  |  |  |  |  |  |  |  |  |  |  |  |  |  |  |  |  |  |  |  |  |  |  |  |  |  |  |  |  |  |  |  |  |  |  |  |  |  |  |  |  |  |  |  |  |  |  |  |  |  |  |  |  |  |  |  |  |  |  |  |  |  |  |  |  |  |  |  |  |  |  |  |  |  |  |  |  |  |  |  |  |  |  |  |  |  |  |  |  |  |  |  |  |  |  |  |  |  |  |  |  |  |  |  |  |  |  |  |  |  |  |  |  |  |  |  |  |  |  |  |  |  |  |  |  |  |  |  |  |  |  |  |  |  |  |  |  |  |  |  |  |  |  |  |  |  |  |  |  |  |  |  |  |  |  |  |  |  |  |  |  |  |  |  |  |  |  |  |  |  |  |  |  |  |  |  |  |  |  |  |  |  |  |  |  |  |  |  |  |  |  |  |  |  |  |  |  |  |
| --- | --- | --- | --- | --- | --- | --- | --- | --- | --- | --- | --- | --- | --- | --- | --- | --- | --- | --- | --- | --- | --- | --- | --- | --- | --- | --- | --- | --- | --- | --- | --- | --- | --- | --- | --- | --- | --- | --- | --- | --- | --- | --- | --- | --- | --- | --- | --- | --- | --- | --- | --- | --- | --- | --- | --- | --- | --- | --- | --- | --- | --- | --- | --- | --- | --- | --- | --- | --- | --- | --- | --- | --- | --- | --- | --- | --- | --- | --- | --- | --- | --- | --- | --- | --- | --- | --- | --- | --- | --- | --- | --- | --- | --- | --- | --- | --- | --- | --- | --- | --- | --- | --- | --- | --- | --- | --- | --- | --- | --- | --- | --- | --- | --- | --- | --- | --- | --- | --- | --- | --- | --- | --- | --- | --- | --- | --- | --- | --- | --- | --- | --- | --- | --- | --- | --- | --- | --- | --- | --- | --- | --- | --- | --- | --- | --- | --- | --- | --- | --- | --- | --- | --- | --- | --- | --- | --- | --- | --- | --- | --- | --- | --- | --- | --- | --- | --- | --- | --- | --- | --- | --- | --- | --- | --- | --- | --- | --- | --- | --- | --- | --- | --- | --- | --- | --- | --- | --- | --- | --- | --- | --- | --- | --- | --- | --- | --- | --- | --- | --- | --- | --- | --- | --- | --- | --- | --- | --- | --- | --- | --- | --- | --- | --- | --- | --- | --- | --- | --- | --- | --- | --- | --- | --- | --- | --- | --- | --- | --- | --- | --- | --- | --- | --- | --- | --- | --- | --- | --- |
| |  |  |  |  |  |  |  |  |  |  |  |  |  |  |  |  |  |  |  | | --- | --- | --- | --- | --- | --- | --- | --- | --- | --- | --- | --- | --- | --- | --- | --- | --- | --- | --- | | |  |  |  |  |  |  |  |  |  |  |  |  |  |  |  |  |  |  | | --- | --- | --- | --- | --- | --- | --- | --- | --- | --- | --- | --- | --- | --- | --- | --- | --- | --- | | |  |  | | --- | --- | | Results | | | Maximum Fit | 13.2912 | | Total Cost | 468.714 | | RMS | 4.99846 | | Correlation | 0.81074 | | |  |  | | --- | --- | | Description | | | Features | Weights | | HBA  HYDROPHOBIC  HYDROPHOBIC  HYDROPHOBIC  HYDROPHOBIC | 2.65824  2.65824  2.65824  2.65824  2.65824 | | | |  | | |  |  |  |  |  |  |  | | --- | --- | --- | --- | --- | --- | --- | | Name | Fit | Est | Act | Err | Status | Mapping | | 4OAS | 13.010 | 0.10000 | 0.1 | 1 | active | [17 3 21 28 5 ] | | 4QOC\_35T\_C\_201 | 12.910 | 0.13000 | 0.1 | 1.3000 | active | [17 3 39 26 5 ] | | 5LN2 | 10.330 | 50 | 0.13 | 390 | active | [\* 15 7 40 19 ] | | 4erf | 12.710 | 0.21000 | 1.1 | -5.3000 | moderately active | [17 3 21 30 5 ] | | 4ZTI | 11.220 | 6.5000 | 1.6 | 4.1000 | moderately active | [30 11 16 20 3 ] | | 4OCC | 12.900 | 0.14000 | 2 | -15 | moderately active | [16 3 33 25 5 ] | | Molecule1 | 10.530 | 32 | 2 | 16 | moderately active | [\* 30 29 32 2 ] | | Molecule2 | 10.400 | 42 | 2 | 21 | moderately active | [\* 24 39 29 2 ] | | Molecule3 | 10.430 | 40 | 4 | 10 | moderately active | [\* 24 31 29 1 ] | | 4LWV | 10.370 | 46 | 7 | 6.6000 | moderately active | [\* 30 4 11 33 ] | | 4OQ3 | 11.190 | 6.9000 | 8 | -1.2000 | moderately active | [23 30 18 1 28 ] | | Molecule4 | 10.410 | 42 | 16 | 2.6000 | moderately active | [\* 24 30 29 2 ] | | 4DIJ | 9.7200 | 200 | 30 | 6.8000 | moderately active | [\* 3 20 2 27 ] | | Molecule5 | 10.370 | 46 | 39 | 1.2000 | moderately active | [\* 27 34 29 1 ] | | 1t4e | 9.8000 | 170 | 80 | 2.1000 | moderately active | [\* 18 3 31 19 ] | | Molecule6 | 10.410 | 42 | 81 | -1.9000 | moderately active | [\* 24 31 29 2 ] | | 4HGF | 9.9200 | 130 | 90 | 1.4000 | moderately active | [6 38 8 14 39 ] | | Molecule7 | 10.530 | 32 | 140 | -4.3000 | moderately active | [\* 27 34 29 2 ] | | 1TTV | 9.7100 | 210 | 160 | 1.3000 | moderately active | [\* 12 27 24 10 ] | | Molecule8 | 10.420 | 41 | 210 | -5.2000 | moderately active | [\* 26 32 28 2 ] | | Molecule9 | 8.7100 | 2100 | 230 | 9.1000 | moderately active | [\* 24 31 26 2 ] | | Molecule10 | 10.360 | 47 | 560 | -12 | inactive | [\* 21 28 27 1 ] | | Molecule11 | 9.8600 | 150 | 820 | -5.5000 | inactive | [\* 21 33 27 2 ] | | Molecule12 | 10.040 | 99 | 1100 | -11 | inactive | [\* 21 28 27 2 ] | | 3JZK | 9.1900 | 690 | 1200 | -1.8000 | inactive | [\* 22 4 7 19 ] | | Molecule13 | 9.1700 | 720 | 2100 | -2.9000 | inactive | [\* 21 26 25 1 ] | | 4LWT | 9.0700 | 920 | 3900 | -4.3000 | inactive | [\* 11 9 22 17 ] | | Molecule14 | 8.9500 | 1200 | 5500 | -4.5000 | inactive | [31 25 \* 26 22 ] | | Molecule15 | 9.2100 | 660 | 18000 | -28 | inactive | [13 26 \* 25 3 ] | | Molecule16 | 8.1600 | 7400 | 36000 | -4.9000 | inactive | [14 26 \* 25 3 ] | |
