# Supplementary material for: An Application of Fit Quality to Screen MDM2/p53 Protein-Protein Interaction Inhibitors
Source: Molecules. 2018 Dec 1;23(12):3174. doi: 10.3390/molecules23123174 (PMC6321222; doi:10.3390/molecules23123174)
Supplement: Supplementary file 1 [file molecules-23-03174-s001.zip › supplemental files/supplemental information.pdf]

Supplemental Information

An application of fit quality to screen MDM2/p53 protein-protein interaction inhibitors

Xin Xue [1,*], Gang Bao, [1,†] Hai-Qing Zhang, [1,†] Ning-Yi Zhao, [2] Yuan Sun, [3], Yue Zhang [1], Xiao-Long Wang [1]

[1] Department of Medicinal Chemistry, Nanjing University of Chinese Medicine, Nanjing 210038, China
[2] Department of Pharmacy, Red Stone Health Industry International Limited, NO.10 Xianlin Street, Nanjing 210038, China
[3] Department of chemistry and biochemistry, The Ohio State University, Columbus, Ohio,43210,USA
† These authors contributed equally to this work.
* Corresponding authors: Address: Nanjing University of Chinese Medicine, Nanjing 210038, China
Tel/Fax: +86 025 85811916. E-mail address: 300203@njucm.edu.cn (Xin Xue)

1. In the first round of virtual screening (VS), the Receptor-Ligand pharmacophore model (RLPH) was generated from 64 crystal complexes extracted from Protein Data Bank.

Table S1 The crystal complexes for Receptor-Ligand pharmacophore model generation.

PDB ID	released data	resolution	PDB ID	released data	resolution
4ZYC	2015.7.22	1.95	3LBL	2010.3.16	1.6
3TU1	2011.11.2	1.6	3IWY	2010.4.21	1.93
4OQ3	2014.4.23	2.3	4IPF	2013.2.20	
6GGN	2018.9.26	2	4OAS	2014.2.19	1.7
5LAV	2016.11.2	1.73	4OCC	2014.4.2	1.8
5LAW	2016.11.2	1.64	4ODE	2014.4.2	1.8
5LAY	2016.11.2	2.71	4ODF	2014.4.2	2.2
5LAZ	2016.11.2	1.66	4OGN	2014.4.2	1.38
3TJ2	2012.9.12	2.1	4OGT	2014.4.2	1.54
4DIJ	2012.5.2	1.9	4OGV	2014.4.2	2.2
5LN2	2016.9.7	1.58	4QOC	2015.5.6	1.7
4ZFI	2016.10.19	2	4WT2	2014.12.3	1.42
4ZGK	2016.10.19	2	5J7F	2017.5.17	2
1RV1	2004.1.20	2.3	5J7G	2017.5.17	185
3U15	2012.6.27	1.8	5OC8	2018.8.22	1.56
3VBG	2012.6.27	2.8	5TRF	2016.11.9	2.1
3VZV	2013.2.6	2.8	5Z02	2018.1.3	1.35
3W69	2013.6.5	1.9	1T4E	2005.2.8	2.6
4ERE	2012.5.23	1.8	2LZG	2012.11.7	
4ERF	2012.5.23	2	3JZK	2009.11.17	2.1
4JRG	2013.7.24	1.9	4HBM	2012.10.17	1.9
4JSC	2013.7.24	2.5	4HG7	2013.7.31	1.6
4JV7	2013.5.1	2.2	4ZYF	2015.7.29	1.8
4JV9	2013.5.1	2.5	4ZY1	2015.7.29	1.67
4JVE	2013.5.1	2.3	5C5A	2016.6.29	1.15
4JVR	2013.5.1	1.7	1TTV	2002.1.1	
4JWR	2013.5.1	2.35	3T4E	2013.4.24	1.91
4WDN	2013.11.13	1.9	4J74	2013.8.7	1.2
4MDQ	2013.11.13	2.12	4J7D	2013.8.7	1.25
4OBA	2014.3.19	1.6	4J7E	2013.8.7	1.63
4QO4	2014.7.16	1.7	4LWT	2014.7.16	1.6
3LBK	2010.3.16	2.3	4LWU	2014.7.16	1.14

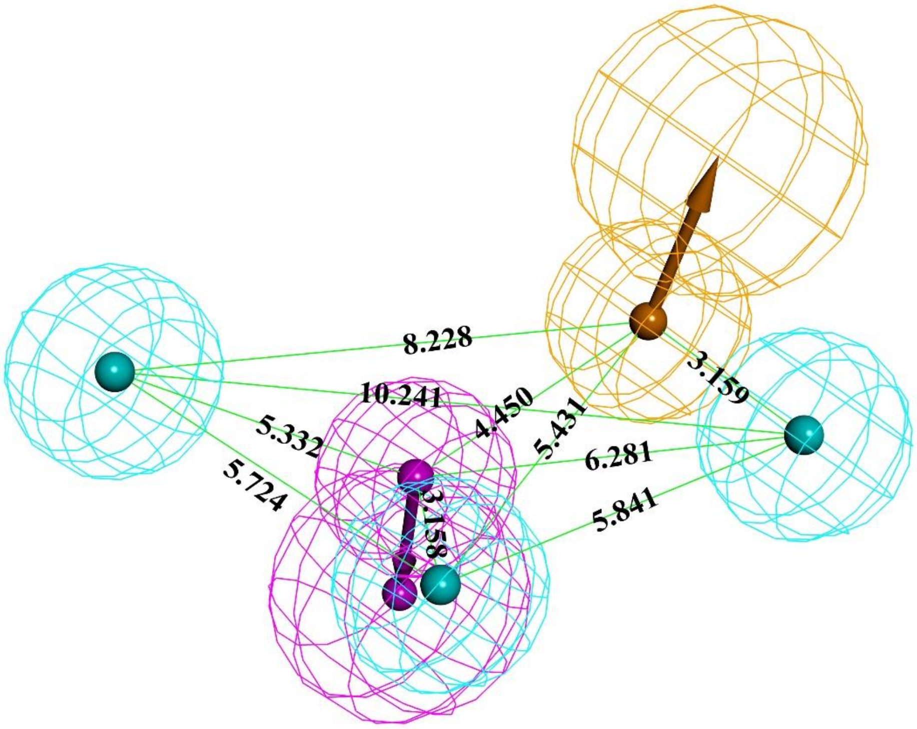

Figure S1. The detail information for Receptor-Ligand pharmacophore model. Purple is a hydrogen bond donor; green is a hydrogen bond acceptor, and cyan is a hydrophobic or an aromatic element.

2. 156 MDM2/p53 Protein-Protein interaction inhibitors (PPIIs) assigned to eight groups were collected from literatures for training and test sets. The heavy atoms (HA) of those compounds were counted to calculate their LE and FQ values. All 156 compounds (**Table S2**) divided in group A-H were used as test set, while compounds tagged with * were used as training set.

Table S2. Eight groups of MDM2/p53 PPIIs were collected for training and test set.

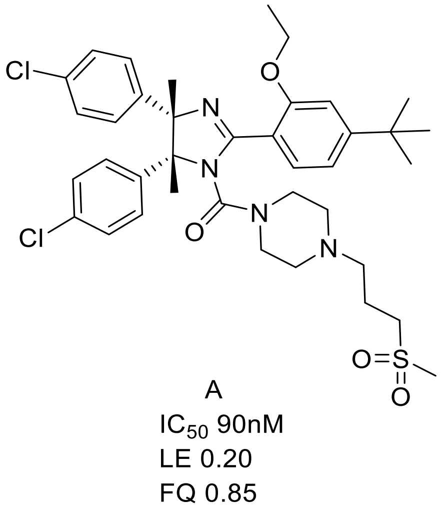

A
IC_{50} 90nM
LE 0.20
FQ 0.85

Name	MDM2/p53 PPIIs		HA	LE	FQ
	IC_{50}/nM	pIC_{50}			

A-1*	52	7.28	43	0.23	0.91
A-2	30	7.52	42	0.25	0.94
A-3*	209	6.68	42	0.22	0.84
A-4	23	7.64	45	0.23	0.94
A-5*	22	7.66	48	0.22	0.92
A-6	26	7.59	45	0.23	0.93
A-7	18	7.74	49	0.22	0.93
A-8	46	7.34	49	0.21	0.88
A-9	33	7.48	48	0.21	0.90
A-10	14	7.85	50	0.22	0.94
A-11	18	7.74	51	0.21	0.92
A-12*	2163	5.66	51	0.15	0.67
A-13	232	6.63	48	0.19	0.80

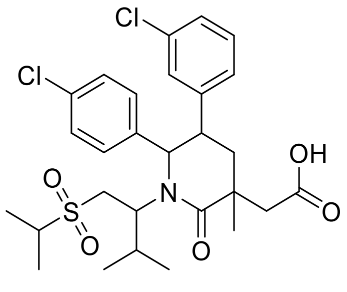

B

IC_{50} 0.1nM

LE 0.37

FQ 1.29

Name	MDM2/p53 PPIIs		HA	LE	FQ
	IC_{50}/nM	pIC_{50}			
B-1	72	7.14	32	0.31	0.95
B-2	47	7.33	44	0.23	0.91
B-3*	3	8.52	36	0.32	1.11
B-4	23	7.64	40	0.26	0.97
B-5	897	6.05	47	0.18	0.73
B-6	547	6.26	42	0.20	0.78
B-7*	14500	4.84	43	0.15	0.60
B-8	33	7.48	40	0.26	0.95
B-9	5	8.30	44	0.26	1.03
B-10	730	6.14	44	0.19	0.76
B-11*	8	8.10	44	0.25	1.00
B-12	6	8.22	45	0.25	1.01
B-13	11	7.96	45	0.24	0.98
B-14	4	8.40	40	0.29	1.06
B-15*	50	7.30	40	0.25	0.93
B-16	4	8.40	40	0.29	1.06
B-17	4	8.40	41	0.28	1.06

B-18	16	7.80	42	0.25	0.98
B-19*	157	6.80	44	0.21	0.84

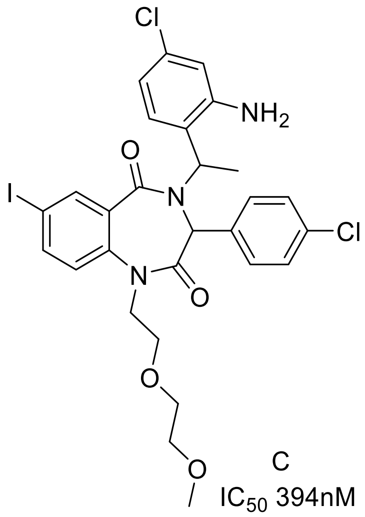

C
IC_{50} 394nM
LE 0.23
FQ 0.82

Name	MDM2/p53 PPIIs		HA	LE	FQ
	IC_{50}/nM	pIC_{50}			
C-1*	0.42	9.38	32	0.40	1.25
C-2	2.18	8.66	33	0.36	1.15
C-3	3.33	8.48	37	0.31	1.09
C-4*	4.35	8.36	39	0.29	1.07
C-5	10.2	7.99	36	0.30	1.04
C-6	1.09	8.96	37	0.33	1.16
C-7	0.51	9.29	39	0.33	1.19
C-8	36	7.44	37	0.28	0.96
C-9	0.54	9.27	33	0.38	1.23
C-10*	0.87	9.06	39	0.32	1.16
C-11	2.82	8.55	40	0.29	1.08
C-12	2.13	8.67	36	0.33	1.13
C-13	8.22	8.09	41	0.27	1.02
C-14	2.15	8.67	39	0.30	1.11
C-15	0.98	9.01	40	0.31	1.14
C-16*	1.04	8.98	41	0.30	1.13
C-17	0.71	9.15	38	0.33	1.17
C-18	1.11	8.95	39	0.31	1.14

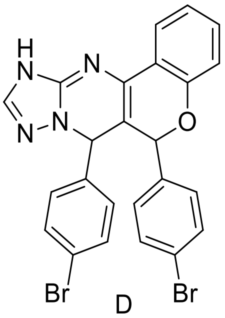

D

IC_{50} 350nM

LE 0.29

FQ 0.87

Name	MDM2/p53 PPIIs		HA	LE	FQ
	IC_{50}/nM	pIC_{50}			
D-1	100000	4.00	31	0.18	0.54
D-2	100000	4.00	31	0.18	0.54
D-3	100000	4.00	31	0.18	0.54
D-4*	14800	4.83	32	0.21	0.64
D-5	1210	5.92	32	0.25	0.79
D-6	1170	5.93	32	0.25	0.79
D-7	6570	5.18	32	0.22	0.69
D-8	890	6.05	32	0.26	0.81
D-9	4630	5.33	32	0.23	0.71
D-10*	2080	5.68	33	0.24	0.75
D-11	2478	5.61	34	0.23	0.74
D-12	9074	5.04	34	0.20	0.66
D-13	100000	4.00	33	0.17	0.53
D-14	1889	5.72	32	0.25	0.76
D-15	4740	5.32	32	0.23	0.71
D-16	5840	5.23	33	0.22	0.69
D-17	1170	5.93	32	0.25	0.79
D-18	1720	5.76	33	0.24	0.76
D-19	360	6.44	34	0.26	0.85
D-20	910	6.04	35	0.24	0.79
D-21	1730	5.76	33	0.24	0.76
D-22	1405	5.85	33	0.24	0.78
D-23	440	6.36	33	0.26	0.84
D-24	3200	5.49	34	0.22	0.72
D-25	3065	5.51	34	0.22	0.73
D-26	990	6.00	33	0.25	0.80
D-27	15100	4.82	33	0.20	0.64
D-28	3500	5.46	33	0.23	0.72
D-29	3940	5.40	35	0.21	0.71
D-30	480	6.32	34	0.25	0.83
D-31	100000	4.00	34	0.16	0.53

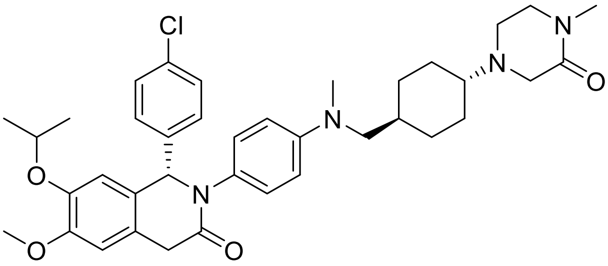

E
IC_{50} 350nM
LE 0.19
FQ 0.78

Name	MDM2/p53 PPIIs		HA	LE	FQ
	IC_{50}/nM	pIC_{50}			
E-1*	3860	5.41	40	0.19	0.69
E-2	1090	5.96	40	0.20	0.76
E-3	2850	5.55	41	0.19	0.70
E-4*	1130	5.95	43	0.19	0.74
E-5	710	6.15	42	0.20	0.77
E-6	14500	4.84	43	0.15	0.60
E-7	4960	5.30	45	0.16	0.65
E-8	350	6.46	45	0.20	0.79
E-9	11300	4.95	46	0.15	0.60
E-10*	5000	5.30	42	0.17	0.66
E-11	13200	4.88	46	0.15	0.60
E-12	20000	4.70	47	0.14	0.57
E-13	20000	4.70	47	0.14	0.57
E-14*	20000	4.70	47	0.14	0.57

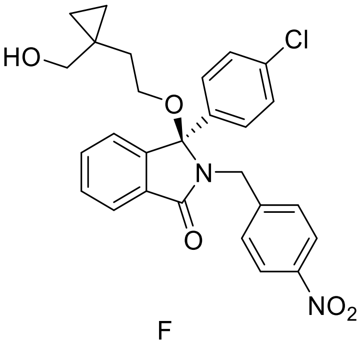

F
IC_{50} 170nM
LE 0.30
FQ 0.89

Name	MDM2/p53 PPIIs		HA	LE	FQ
	IC_{50}/nM	pIC_{50}			
F-1	90000	4.05	33	0.17	0.54
F-2	85000	4.07	33	0.17	0.54
F-3	27000	4.57	41	0.15	0.58

F-4*	66000	4.18	31	0.18	0.56
F-5	70000	4.15	34	0.17	0.55
F-6	284000	3.55	34	0.14	0.47
F-7	393000	3.41	31	0.15	0.46
F-8	78000	4.11	35	0.16	0.54
F-9	311000	3.51	36	0.13	0.46
F-10	58000	4.24	32	0.18	0.57
F-11	96000	4.02	38	0.14	0.52
F-12	243000	3.61	32	0.15	0.48
F-13	345000	3.46	30	0.16	0.47
F-14	490000	3.31	28	0.16	0.45
F-15	500000	3.30	29	0.16	0.45
F-16	181000	3.74	34	0.15	0.49
F-17	413000	3.38	29	0.16	0.46
F-18*	88000	4.06	40	0.14	0.51
F-19	500000	3.30	24	0.19	0.47
F-20	70000	4.15	29	0.20	0.57
F-21	70200	4.15	37	0.15	0.54
F-22	103000	3.99	43	0.13	0.50
F-23	100000	4.00	36	0.15	0.52

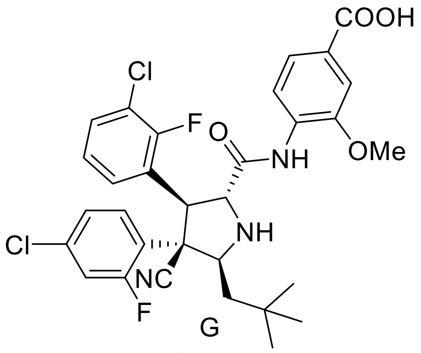

G
IC_{50} 6nM
LE 0.26
FQ 1.03

Name	MDM2/p53 PPIIs		HA	LE	FQ
	IC_{50}/nM	pIC_{50}			
G-1*	196	6.71	35	0.26	0.88
G-2	74	7.13	37	0.26	0.92
G-3	56	7.25	41	0.24	0.91
G-4*	42	7.38	40	0.25	0.93
G-5	23	7.64	40	0.26	0.97
G-6	22	7.66	44	0.24	0.95
G-7	20	7.70	42	0.25	0.96
G-8*	21	7.68	41	0.26	0.97
G-9	25	7.60	41	0.25	0.96
G-10*	6	8.22	42	0.27	1.03

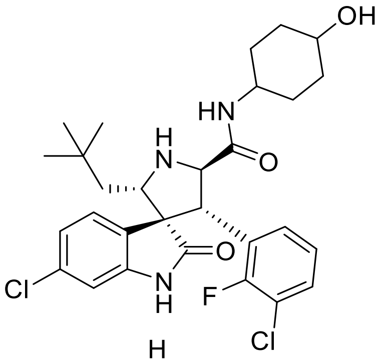

H
IC_{50} 214nM
LE 0.28
FQ 0.86

Name	MDM2/p53 PPIIs		HA	LE	FQ
	IC_{50}/nM	pIC_{50}			
H-1	819	6.09	29	0.29	0.83
H-2	1075	5.97	28	0.29	0.82
H-3	563	6.25	39	0.22	0.80
H-4*	157	6.80	26	0.36	0.95
H-5	2064	5.69	28	0.28	0.78
H-6	57	7.24	29	0.34	0.99
H-7	36500	4.44	28	0.22	0.61
H-8	40100	4.40	29	0.21	0.60
H-9*	15600	4.81	33	0.20	0.64
H-10	18300	4.74	35	0.19	0.62
H-11	5500	5.26	24	0.30	0.74
H-12	15200	4.82	29	0.23	0.66
H-13	30700	4.51	32	0.19	0.60
H-14	16000	4.80	28	0.23	0.66
H-15	13500	4.87	28	0.24	0.67
H-16	157	6.80	25	0.37	0.95
H-17	229	6.64	26	0.35	0.92
H-18*	62	7.21	27	0.37	1.00
H-19	107	6.97	32	0.30	0.93
H-20*	137	6.86	33	0.28	0.91
H-21	212	6.67	38	0.24	0.86
H-22	39	7.41	31	0.33	0.99
H-23	2	8.70	32	0.37	1.16
H-24	80	7.10	31	0.31	0.95
H-25	81	7.09	38	0.26	0.91
H-26	4	8.40	38	0.30	1.08
H-27	16	7.80	38	0.28	1.00
H-28*	2	8.70	44	0.27	1.08

3. The first round of VS was performed by using RLPH with the FitValue ranked.

Table S3. Validation results of the hit identification rate (higher EF) at all levels of screening threshold for constructed Receptor-Ligand pharmacophore model.

**Parameter**	**PH screening method**				
Screening Threshold	0.5%	1%	1.5%	2%	2.5%
a	13	30	41	50	61
n	17	34	51	68	86
A	466	466	466	466	466
N	3418	3418	3418	3418	3418
EF	5.61	6.47	5.90	5.39	5.20

Table S4. 1% out of the library, 335 compounds, passed the first VS by Receptor-Ligand pharmacophore model from NCI and SPECS databases.

Index	NAME/IDNUMBER	MW	HA	FitValue
1	ZINC04390289	359.42	27	3.65
2	ZINC02178970	439.56	32	3.41
3	ZINC08648874	489.36	32	3.29
4	F2692-0095	499.58	36	3.82
5	F2029-0601	458.55	31	3.61
6	F0580-0392	456.52	33	3.59
7	F0580-0398	470.54	34	3.58
8	F0570-0057	470.61	32	3.56
9	F0580-0397	470.54	34	3.52
10	F0580-0403	484.57	35	3.44
11	F1607-1058	491.95	34	3.42
12	F2692-0077	485.55	35	3.33
13	F1607-0980	491.95	34	3.27
14	ZINC01027435	505.92	32	5.16
15	ZINC01027072	475.43	31	4.78
16	ZINC08637136	493.61	33	3.83
17	ZINC04297060	398.47	28	3.51
18	ZINC02142855	490.32	31	3.49
19	ZINC03046141	388.29	26	3.39
20	ZINC01027046	491.89	31	3.34
21	ZINC03185768	417.56	30	3.13
22	ZINC03185778	381.88	26	3.03
23	STOCK1N-31983	582.67	42	4.52
24	STOCK1N-36517	483.95	35	4.18
25	STOCK1N-34721	509.55	38	4.15
26	STOCK1N-34937	614.64	46	4.13

27	STOCK1N-32729	665.53	45	4.11
28	STOCK1N-33413	649.49	44	4.1
29	STOCK1N-33491	665.53	45	4.08
30	STOCK1N-33053	595.44	40	4.04
31	STOCK1N-33483	635.5	43	4.04
32	STOCK1N-35012	496.94	36	3.94
33	STOCK1N-40071	534.65	40	3.86
34	STOCK1N-35519	492.52	37	3.74
35	STOCK1N-33261	639.92	42	3.7
36	STOCK1N-33516	684.37	42	3.7
37	STOCK1N-32961	605.47	41	3.59
38	STOCK1N-32687	635.5	43	3.57
39	STOCK1N-29646	571.62	43	3.52
40	STOCK1N-32878	444.52	33	3.46
41	STOCK1N-38179	506.59	38	3.44
42	STOCK1N-31577	498.96	36	3.43
43	STOCK1N-33204	605.48	41	3.38
44	STOCK1N-39437	479.53	36	3.32
45	STOCK1N-33649	675.61	46	3.27
46	STOCK1N-32758	527.57	40	3.26
47	STOCK1N-30511	482.5	36	3.26
48	STOCK1N-48215	540.61	41	3.24
49	STOCK1N-29715	498.96	36	3.21
50	STOCK1N-22270	518.65	39	3.2
51	STOCK1N-33428	414.5	31	3.15
52	STOCK1N-36313	494.5	37	3.11
53	STOCK1N-02317	573.7	41	3.06
54	N-(4,6-dimethyl-2-pyrimidinyl)-N'-(4-ethylphenyl)-N"-(10H-phenothiazi	494.61	36	4.04
55	N-(4,6-dimethyl-2-pyrimidinyl)-N'-(4-methoxyphenyl)-N"-(10H-phenothia	496.58	36	3.95
56	11-(2,4,5-trimethoxyphenyl)-3-(3,4,5-trimethoxyphenyl)-2,3,4,5,10,11-h	546.61	40	3.73
57	11-[4-(methylsulfanyl)phenyl]-3-(3,4,5-trimethoxyphenyl)-2,3,4,5,10,11	502.63	36	3.39
58	N-(4-chlorophenyl)-N'-(4,6-dimethyl-2-pyrimidinyl)-N"-(10H-phenothiaz	501	35	3.38

59	11-(5-methyl-2-thienyl)-3-(3,4,5-trimethoxyphenyl)-2,3,4,5,10,11-hexah	476.59	34	3.33
60	AF-399	379.34	26	3.31
61	11-(1,3-benzodioxol-5-yl)-3-(3,4,5-trimethoxyphenyl)-2,3,4,5,10,11-hex	500.54	37	3.26
62	11-(3-bromo-4,5-dimethoxyphenyl)-3-(3,4,5-trimethoxyphenyl)-2,3,4,5,10	595.48	39	3.26
63	1-(2,3-diphenyl-1H-benzo[g]indol-1-yl)-3-(4,5-diphenyl-1H-imidazol-1-y	595.73	46	3.24
64	1-(5,7-dimethyl-2,3-diphenyl-1H-indol-1-yl)-3-(4,5-diphenyl-1H-imidazo	573.73	44	3.13
65	11-(3-fluorophenyl)-10-(trifluoroacetyl)-3-(3,4,5-trimethoxyphenyl)-2,	570.53	41	3.12
66	NSC 13630	464.86	30	4.11
67	NSC 49789	438.61	28	3.63
68	NSC 18211	383.52	28	3.63
69	NSC 69184	376.4	28	3.56
70	NSC 18207	355.47	26	3.45
71	NSC 36506	446.5	33	3.31
72	NSC 71214	432.7	26	3.29
73	NSC 84095	355.43	27	3.24
74	NSC 99550	385.5	30	3.21
75	NSC 22535	598.78	46	3.18
76	NSC 69899	923.01	68	3.16
77	SPB 05822	388.29	26	3.02
78	ZINC70687027	540.61	41	4.3
79	ZINC70687439	540.61	41	4.22
80	ZINC70686506	562.84	36	3.83
81	ZINC70686851	584.62	44	3.67
82	ZINC20756120	490.49	36	3.62
83	ZINC70686508	562.84	36	3.61
84	ZINC70687674	556.61	42	3.56
85	ZINC70687669	527.57	40	3.48
86	ZINC59485880	507.58	38	3.45
87	ZINC12880882	490.49	36	3.42
88	ZINC35363850	513.97	37	3.42
89	ZINC70706882	550.61	39	3.37

90	ZINC70687722	570.63	43	3.35
91	ZINC02109854	539.62	40	3.33
92	ZINC15968695	519.57	38	3.29
93	ZINC70687660	584.66	44	3.26
94	ZINC70686793	511.57	39	3.21
95	ZINC70686605	532.67	40	3.18
96	ZINC70705922	571.62	42	3.17
97	ZINC70706887	553.65	39	3.16
98	ZINC70687774	556.61	42	3.13
99	ZINC70706625	558.61	41	3.08
100	ZINC70687338	541.6	41	3.04
101	ZINC70706121	555.6	41	3.03
102	ZINC11866307	457.52	34	3.31
103	ZINC31166474	412.43	30	4.22
104	ZINC08635519	497.63	35	4.21
105	ZINC03839167	507.58	37	3.73
106	ZINC35415844-2	447.57	33	3.17
107	STOCK1N-30600	435.47	33	3
108	2-(2-methylphenoxy)-N-(2- [(2-methylphenoxy)acetyl]amino phenyl)acetam	404.46	30	3.01
109	ZINC70687869	584.66	44	3.02
110	ZINC70687857	540.61	41	3
111	ZINC70706946	569.63	42	3
112	NSC 1049	376.06	17	3.36
113	NSC 2855	330.48	23	3.32
114	NSC 5460	381.16	24	3.24
115	NSC 391	324.2	21	3.23
116	NSC 7215	256.35	19	3.19
117	NSC 1050	408.06	19	3.14
118	NSC 4924	327.36	23	3.13
119	NSC 5885	279.34	21	3.11
120	NSC 7871	385.25	26	3.1
121	NSC 4479	356.43	23	3.08
122	NSC 3237	422.58	27	3.07
123	NSC 683	284.42	20	3.04
124	NSC 8360	480.02	34	3
125	NSC 69898	838.94	62	3.48
126	NSC 84095	355.43	27	3.24
127	HTS 00131	459.49	33	3.64
128	SPB 05646	304.3	22	3.5
129	RJF 00940	348.85	23	3.47

130	BTB 04860	426.22	28	3.41
131	KM 01222	476.78	28	3.34
132	SPB 05822	388.29	26	3.3
133	BTB 04175	379.92	25	3.14
134	BTB 06517	339.36	23	3.04
135	GK 01508	510.31	35	3.04
136	RH 01670	447.5	30	3.93
137	RH 01679	369.55	25	3.9
138	BTB 05674	441.45	30	3.81
139	RH 01967	366.84	26	3.51
140	RJF 01798	303.34	22	3.48
141	NSC 985	317.26	18	3.58
142	NSC 308	499.82	18	3.47
143	NSC 5844	383.27	26	3.46
144	NSC 364	281.13	18	3.42
145	NSC 3753	304.36	21	3.38
146	NSC 1014	341.41	26	3.33
147	NSC 4112	287.16	17	3.36
148	AE-562	359.53	25	3.61
149	ZINC05217831	387.43	28	4.17
150	ZINC04157245	359.8	25	3.94
151	ZINC09594416	432.31	27	3.5
152	ZINC01112039	355.83	23	3.44
153	ZINC08439189	443.53	30	3.31
154	ZINC08439200	435.92	29	3.24
155	ZINC00119495	323.76	23	3.2
156	ZINC35830493	443.41	32	3.18
157	ZINC17968970	386.43	27	3.66
158	ZINC00632474	393.83	27	3.14
159	ZINC02483499	481.56	34	3.12
160	ZINC00687864	394.44	27	3.07
161	ZINC00684653	388.26	24	3.06
162	ZINC04707806	309.75	22	3.68
163	ZINC13597219	301.19	18	3.64
164	ZINC04823467	399.34	28	3.62
165	ZINC13597762	245.23	18	3.59
166	ZINC04707806	309.75	22	3.59
167	ZINC13152247	462.14	25	3.55
168	ZINC17949075	401.22	27	3.52
169	ZINC13130011	253.26	19	3.48
170	ZINC00138096	332.87	21	3.48
171	ZINC01574971	284.20	17	3.47

172	ZINC17858074	359.26	22	3.46
173	ZINC15990251	286.33	21	3.43
174	ZINC18098743	388.47	29	3.43
175	ZINC13597219	301.19	18	3.41
176	ZINC17968970	386.43	27	3.38
177	ZINC15990220	244.27	18	3.37
178	ZINC15990251	287.34	21	3.33
179	ZINC01045051	367.25	23	3.32
180	ZINC00002036	238.29	18	3.31
181	ZINC13597758	249.65	17	3.31
182	ZINC01649010	295.4	21	3.26
183	ZINC00163657	258.27	19	3.23
184	ZINC04707664	451.11	24	3.22
185	ZINC18153859	293.12	17	3.22
186	ZINC18219562	346.83	23	3.17
187	ZINC01686467	329.78	23	3.17
188	ZINC17858074	359.26	22	3.13
189	ZINC05640410	292.29	19	3.09
190	ZINC01718486	385.25	26	3.07
191	ZINC08581317	304.36	21	3.06
192	STOCK1N-04142	428.32	27	4.66
193	STOCK1N-05707	324.37	24	4.6
194	STOCK1N-00355	379.41	28	4.5
195	STOCK1N-07078	325.32	24	4.43
196	STOCK1N-03083	328.36	24	4.43
197	STOCK1N-02095	340.37	25	4.42
198	STOCK1N-06661	366.41	27	4.39
199	STOCK1N-01378	366.41	27	4.38
200	STOCK1N-01610	381.42	28	4.37
201	STOCK1N-06501	338.36	25	4.37
202	STOCK1N-06154	394.46	29	4.33
203	STOCK1N-00242	342.39	25	4.32
204	STOCK1N-00322	352.38	26	4.27
205	STOCK1N-03781	342.39	25	4.18
206	STOCK1N-01777	310.34	23	4.18
207	STOCK1N-05528	291.35	22	3.68
208	STOCK1N-04034	386.44	28	3.46
209	STOCK1N-04873	396.39	29	3.38
210	STOCK1N-03803	357.36	26	3.34
211	STOCK1N-03839	327.37	24	3.31
212	STOCK1N-03575	310.35	23	3.24
213	STOCK1N-05640	374.43	27	3.11

214	STOCK1N-02028	309.36	23	3.10
215	STOCK1N-04641	309.36	23	3.05
216	STOCK1N-00384	360.45	27	3.05
217	F0020-0031	313.37	22	3.44
218	F0372-0505	299.33	22	3.23
219	F0049-0016	276.35	19	3.14
220	ZINC33382220	286.72	20	3.77
221	ZINC03170843	446.71	27	3.70
222	ZINC33382264	383.68	25	3.48
223	ZINC20413478	475.37	34	3.31
224	ZINC33382250	334.22	23	3.23
225	ZINC12367813	349.43	26	3.22
226	ZINC01038224	370.27	24	3.17
227	ZINC01025111	363.45	24	3.12
228	ZINC02161072	463.76	31	3.05
229	ZINC24044552	294.38	20	4.06
230	ZINC48962820	301.15	19	3.97
231	ZINC45025350	284.77	18	3.33
232	ZINC48893551	279.72	19	3.24
233	ZINC48962858	316.18	19	3.16
234	ZINC00126828	302.42	20	3.72
235	ZINC01033175	454.39	30	3.18
236	ZINC04956632	396.26	23	3.17
237	ZINC12367828	491.97	35	3.11
238	ZINC00126829	322.84	20	3.11
239	ZINC01324022	393.44	29	4.71
240	ZINC03984097	352.38	26	3.48
241	ZINC20762726	437.49	32	3.36
242	ZINC08917944	382.41	28	3.35
243	ZINC20762588	437.49	32	3.30
244	ZINC00619547	292.33	22	3.08
245	ZINC05489491	352.38	26	4.64
246	ZINC13127397	340.37	25	4.34
247	ZINC18167494	312.32	23	4.33
248	ZINC05452024	325.36	24	4.27
249	ZINC05451848	311.33	23	4.23
250	ZINC05197560	354.83	25	4.20
251	ZINC13121705	385.25	24	4.19
252	ZINC18098944	336.38	25	4.10
253	ZINC08740587	352.38	26	4.10
254	ZINC18091290	366.41	27	3.99
255	ZINC02328428	501.89	35	3.96

256	ZINC13127250	326.35	24	3.90
257	ZINC08440369	445.73	29	3.85
258	ZINC09058845	451.90	32	3.85
259	ZINC13126250	314.77	22	3.84
260	ZINC05451863	315.75	22	3.84
261	ZINC05451687	369.72	25	3.83
262	ZINC05191626	354.36	26	3.74
263	ZINC09187792	450.91	32	3.66
264	ZINC09042841	466.45	34	3.58
265	ZINC09130707	458.51	34	3.58
266	ZINC09129721	471.33	32	3.55
267	ZINC18251473	420.43	31	3.47
268	ZINC02331744	443.49	33	3.47
269	ZINC05196960	402.44	30	3.44
270	ZINC18114979	348.40	26	3.43
271	ZINC18143398	372.42	28	3.40
272	ZINC05197609	402.44	30	3.39
273	ZINC08973452	404.43	30	3.38
274	ZINC09129204	435.31	28	3.35
275	ZINC02119473	397.38	29	3.33
276	ZINC02120006	309.36	23	3.31
277	ZINC09346403	441.48	33	3.31
278	ZINC41359251	418.44	31	3.29
279	ZINC09346242	447.46	33	3.27
280	ZINC09058986	455.51	34	3.24
281	ZINC05164064	416.47	31	3.22
282	ZINC03985172	338.36	25	3.11
283	ZINC09058967	434.87	31	3.11
284	ZINC05190694	402.44	30	3.09
285	ZINC08024779	321.37	22	3.84
286	ZINC20156590	321.37	22	3.32
287	ZINC01926850	296.36	22	3.26
288	ZINC02526659	275.37	19	3.24
289	ZINC49453363	312.36	22	3.22
290	ZINC34685252	366.41	27	3.08
291	ZINC35457681	452.45	32	3.50
292	ZINC35457671	452.45	32	3.47
293	ZINC03874886	516.54	38	3.42
294	ZINC35457676	452.45	32	3.10
295	ZINC05999108	344.40	25	3.06
296	ZINC31156952	288.34	21	3.82
297	ZINC13383570	356.41	26	3.65

298	ZINC13340317	288.34	21	3.54
299	ZINC35271371	482.48	34	3.52
300	ZINC35271359	482.48	34	3.34
301	ZINC33830232	358.38	26	3.26
302	ZINC31158018	406.43	29	3.24
303	ZINC01595957	358.39	26	3.19
304	ZINC13375623	319.29	23	3.18
305	ZINC49605558	732.86	53	3.16
306	ZINC35465689	405.42	29	3.13
307	ZINC35465687	405.42	29	3.10
308	ZINC31158028	406.43	29	3.08
309	BTB 03180	421.72	28	4.23
310	CD 05733	551.25	35	3.14
311	BTB 08566	214.26	16	3.44
312	NSC 2519	278.33	21	4.33
313	NSC 2262	314.42	23	3.41
314	AA-504/33806053	392.86	27	3.49
315	AA-516/30011006	326.80	22	3.20
316	ZINC06576323	326.80	22	3.37
317	ZINC48962834	286.13	18	3.29
318	ZINC05514370	386.44	28	3.46
319	ZINC41358977	468.89	33	3.64
320	ZINC09129643	428.48	32	3.59
321	ZINC35457628	406.43	29	3.43
322	ZINC67912004	543.50	39	3. 90
323	ZINC49605557	732.86	53	3.28
324	ZINC49605559	732.85	53	3.25
325	ZINC20228213	368.24	22	3.02
326	ZINC48962842	290.72	20	3.00
327	ZINC02098902	438.47	32	3.04
328	ZINC05248942	269.27	20	3.04
329	ZINC05451566	295.33	22	3.03
330	ZINC32919269	325.33	22	3.02
331	ZINC00052541	317.18	19	3.01
332	ZINC35457667	452.45	32	3.04
333	ZINC35457623	406.43	29	3.03
334	ZINC67903416	538.54	38	3.03
335	ZINC14773836	412.56	30	3.01

4. The second round of VS was performed by ligand efficiency (LE) based metric, fit quality (FQ). The HA of 335 compounds were counted, meanwhile the predicted activities, pIC_{50}, were generated by 3D-QSAR pharmacophore model, so that the LE and FQ values could be calculated. 3D-QSAR

pharmacophore model was generated from molecules in training set (Table S2) by employing 3D QSAR Pharmacophore Generation module within Discovery Studio.

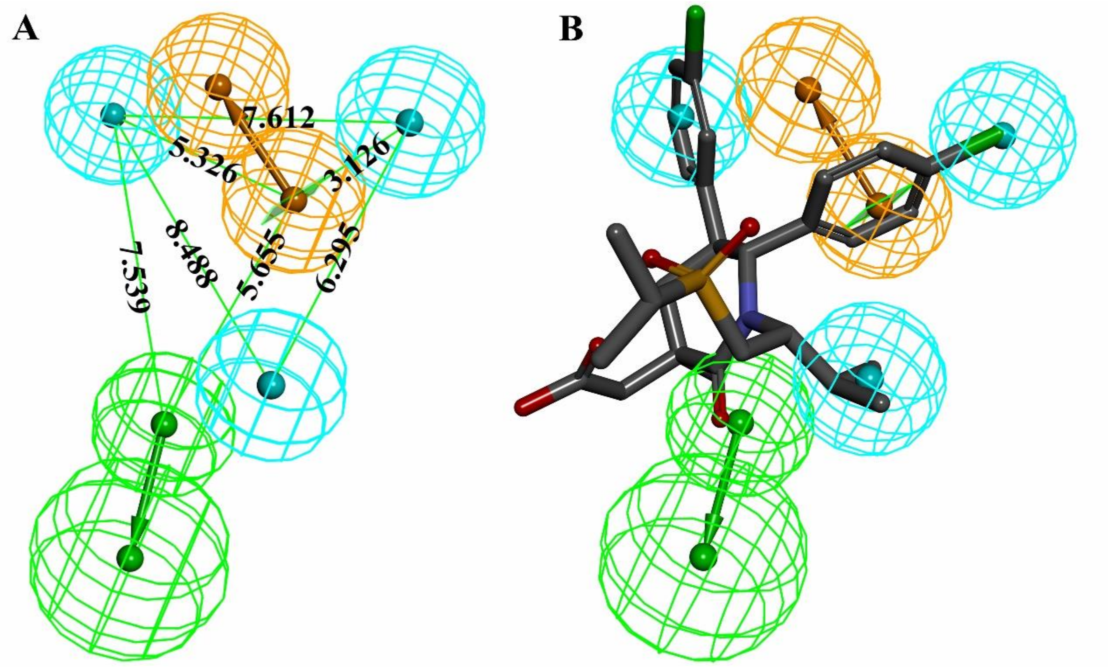

Figure S2. (A) The detail information for 3D-QSAR pharmacophore model. (B) The clinical candidate, **AMG232**, was docked into the 3D-QSAR pharmacophore model.

Table S5. All 26 compounds with the ranked FQ values (FQ>0.8) were screened in the second VS.

Name	Estimate(nM)	pIC50	LE	FQ
AA-504	3.73	8.42	0.43	1.16
STOCK1N-31983	3.66	8.44	0.28	1.06
STOCK1N-33491	3.48	8.46	0.26	1.04
STOCK1N-33649	3.63	8.44	0.25	1.03
ZINC70705922	7.44	8.13	0.28	1.02
ZINC090594416	60.85	7.22	0.37	1.00
ZINC09130707	27.13	7.57	0.30	1.00
STOCK1N-32687	10.85	7.96	0.26	0.99
ZINC8385603	72.80	7.14	0.35	0.98
ZINC02331744	37.48	7.43	0.31	0.98
ZINC59485880	23.13	7.64	0.28	0.98
ZINC01027435	62.61	7.20	0.31	0.96
ZINC70686605	26.15	7.58	0.26	0.96
ZINC01027046	143.88	6.84	0.30	0.92
ZINC05164064	206.06	6.69	0.30	0.90
ZINC31158028	305.12	6.52	0.31	0.89
ZINC05196960	274.95	6.56	0.30	0.89
ZINC09058986	242.80	6.61	0.27	0.87
ZINC00687864	736.98	6.14	0.31	0.85
ZINC41358977	393.24	6.41	0.27	0.85
F2029-0601	551.99	6.26	0.28	0.84

ZINC02142855	579.98	6.24	0.28	0.84
ZINC09346403	433.47	6.36	0.26	0.84
NSC 18211	1257.71	5.90	0.29	0.81
BTB 05674	1247.28	5.90	0.27	0.80
ZINC09346242	876.52	6.06	0.25	0.80

5. Bioassay

In vitro antitumor activity.

Liver hepatocellular carcinoma HepG2 (with wild-type p53) and Hep3B (with p53 null) cell lines were cultured in RPMI 1640 medium containing 10% fetal bovine serum (Gemini Bioproducts) at 37°C in a humidified 5% carbon dioxide humidified incubator.

5-6×10^4 cells per well were plated in 96-well plates (Costar) for 24 h, and the test compounds (prepared in 100% DMSO as a stock solution) were added in triplicate (100μL per well). 8 different concentrations (from 0.05 to 100μM) were tested for each compound. For each assay, the controls included only the medium (blank) and only the cells (positive). After 72 h of incubation, 20 μL of MTT (3-[4,5-dimethylthiazol-2-yl]-2,5-diphenyltetrazoliumbromide) solution (5 mg/mL) was added to each well, and after the samples were shaken for 1 min the plate was incubated further for 4 h at 37°C. Thiobenzodiazepines were dissolved with 150 μL of DMSO and the plates were read using Eon (BioTek) at 570 nm. The IC_{50} was then analyzed using GraphPad Prism software.

The cellular growth inhibitory activity was determined using two human osteosarcoma cell lines, HCT116 $p53^{+/+}$ (with wild-type p53) and HCT116 $p53^{-/-}$ (with p53 deleted, a gift from Dr. Bert Vogelstein, Johns Hopkins University). Cells were cultured in RPMI 1640 medium containing 10% fetal bovine serum (Gemini Bioproducts) at 37°C in a humidified 5% carbon dioxide humidified incubator. 5-6×10^4 cells per well were plated in 96-well plates (Costar) for 24 h, and the test compounds (prepared in 100% DMSO as a stock solution) were added in quadruplicate (100μL per well). 10 different concentrations (from 0 to 100μM) were tested for each compound. For each assay, the controls included only the medium (blank) and only the cells (positive). After 72 h of incubation, 20 μL of MTT (3-[4,5-dimethylthiazol-2-yl]-2,5-diphenyltetrazoliumbromide) solution (5 mg/mL) was added to each well, and after the samples were shaken for 1 min the plate was incubated further for 4 h at 37°C. Thiobenzodiazepines were dissolved with 150 μL of DMSO and the plates were read using Eon (BioTek) at 570 nm. The IC_{50} was then analyzed using GraphPad Prism software.

Fluorescence Polarization Binding Assay.

The compounds identified as possible MDM2 inhibitors were purchased from NCI Database. For testing their binding affinities to MDM2 protein, we performed a sensitive and quantitative FP-based binding assay[1, 2] using human recombinant His-fused soluble protein MDM2 (residues 1 -118) and a p53-based peptide labeled with a fluorescence tag, termed as PMDM6-F (Anaspec, 10nM). The K_d value of PMDM6-F with the MDM2 protein was determined to be 3.30 ± 0.12 nM. The fluorescence experiments were performed as described in the literature.[3] Briefly, the fluorescence polarization experiments were read on SpectraMax Paradigm Multi-mode Detection Platform (Molecular Devices) with the 485 nm excitation and 535 nm emission filters. The fluorescence intensities parallel (Intparallel) and perpendicular (Intperpedicular) to the plane of excitation were measured in black 96-well NBS assay plates (Greiner Microlon) at room temperature. The background fluorescence intensities of blank samples containing the reference buffer were subtracted, and steady-state fluorescence polarization was calculated using the following equation: P = 1000× (Intparallel −

GIntperpedicular)/ (Intparallel + GIntperpedicular), and the correction factor G (G = 0.998 determined empirically) was introduced to eliminate differences in the transmission of vertically and horizontally polarized light. All fluorescence polarization (FP) values were expressed in millipolarization units (mP). The dose-dependent binding experiments were carried out with serial dilutions (from 0 to 1000 μM) of the tested compounds in DMSO. A 50 μL sample of the tested samples and preincubated MDM2 protein (10 nM) and PMDM6-F peptide (10 nM) in the assay buffer (100 mM potassium phosphate, pH 7.5; 100 μg/mL bovine gamma globulin; 0.02% sodium azide) were added in the black 96-well NBS assay plates (Greiner Microlon) to produce a final volume of 125 μL. For each assay, the controls included the MDM2 protein and PMDM6-F (equivalent to 0% inhibition) and only the PMDM6-F peptide (equivalent to 100% inhibition). The polarization values were measured after 0.5h of the mixing of all assay components. Binding constant (K_i) and inhibition curves were fitted using GraphPad Prism software and a web-based computer program developed by Wang.[4]

6. The chemical structures, the curve of inhibitory and the binding modes of the six hits.

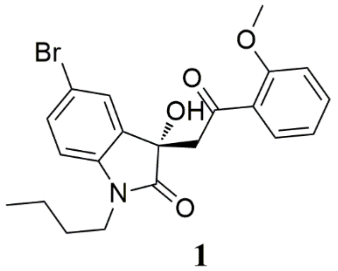

1
ZINC09594416

Figure S3 the structure of **ZINC09594416**

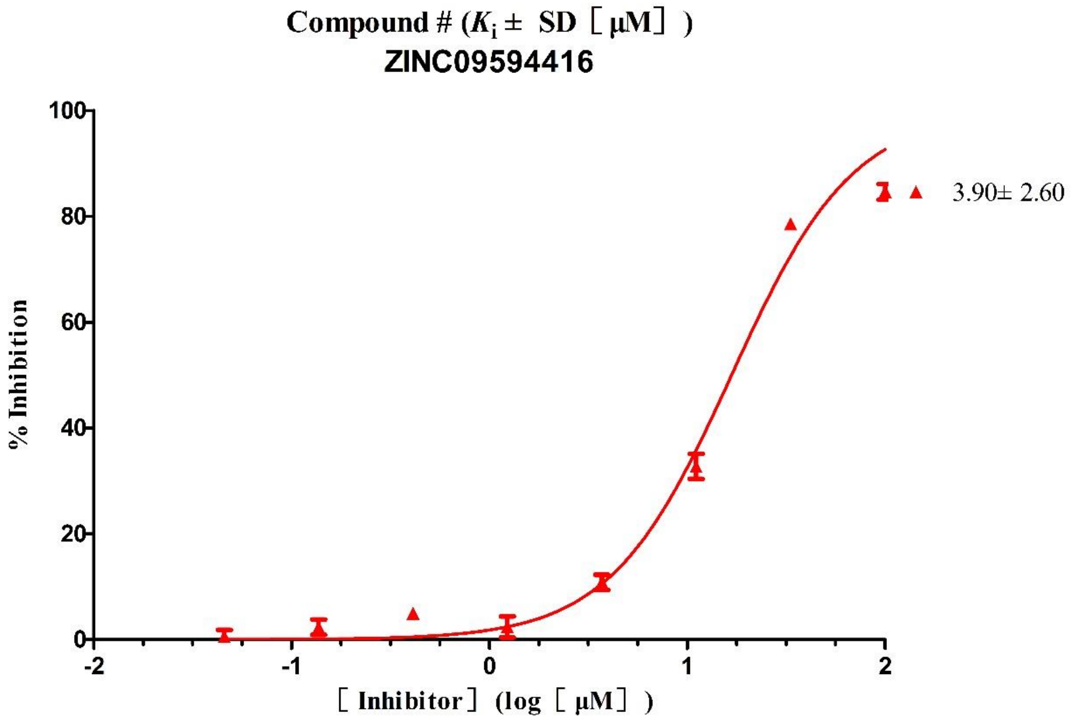

Figure S4 the curve of inhibitory of **ZINC09594416**

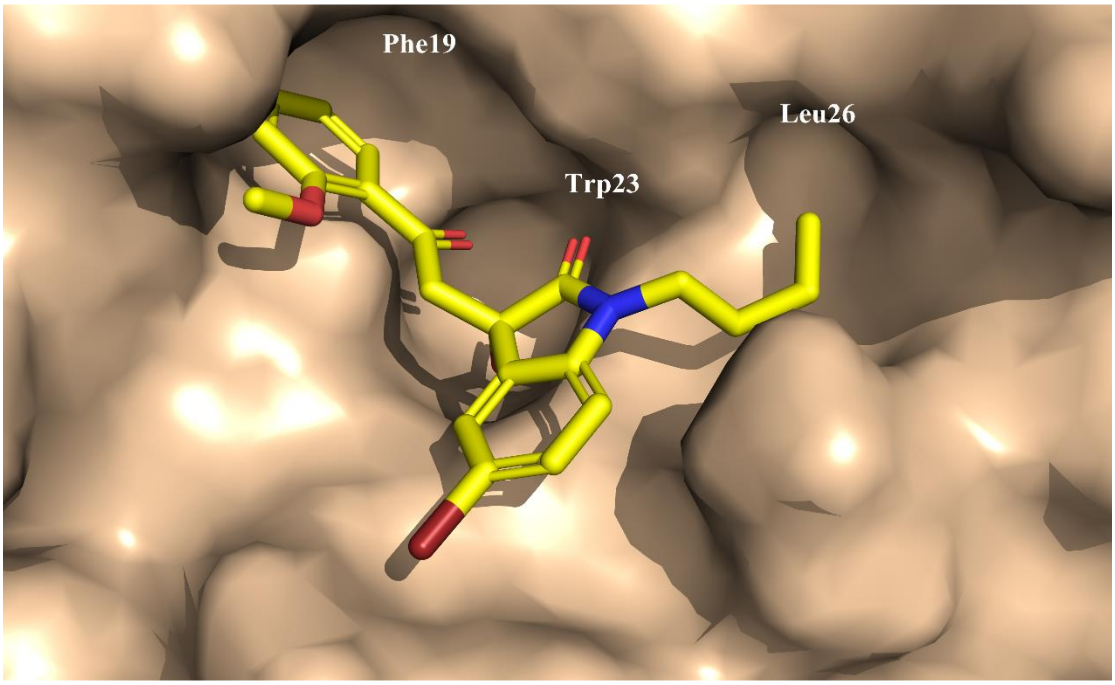

Figure S5 Predicted binding modes of **ZINC09594416** to MDM2. The protein displayed as a gray surface and its key residues were shown with labels. All compounds are shown with only backbone atoms.

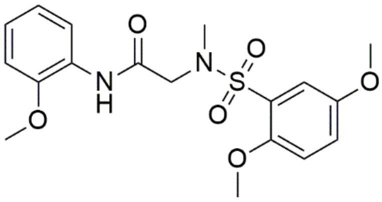

2

ZINC00687864

Figure S6 the structure of **ZINC00687864**

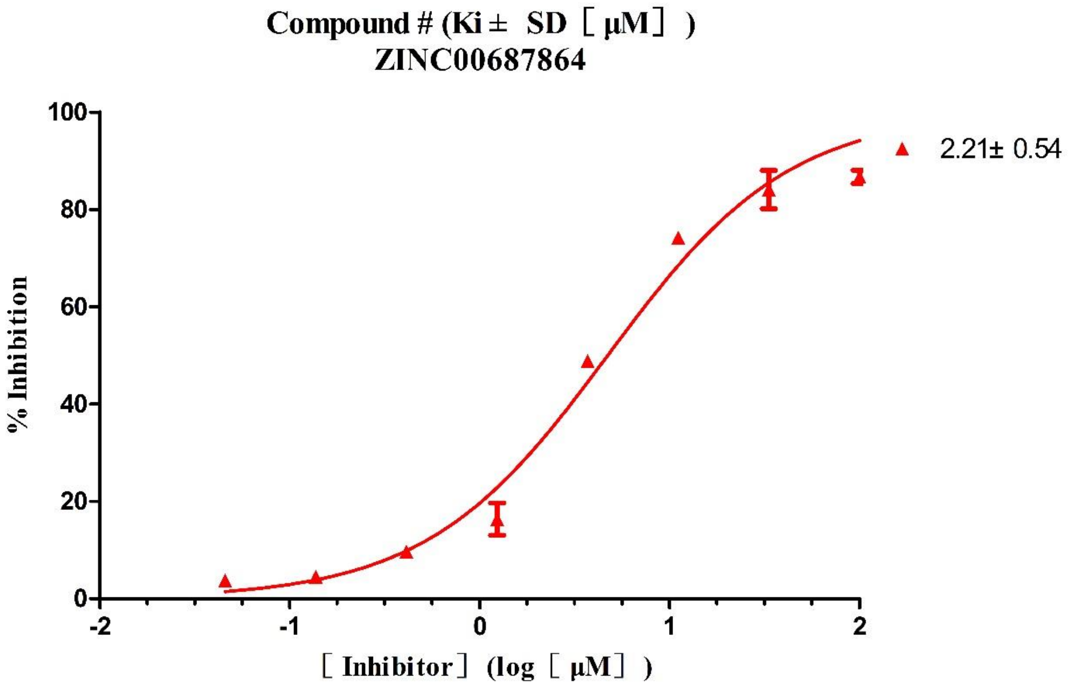

Figure S7 the curve of inhibitory of **ZINC00687864**

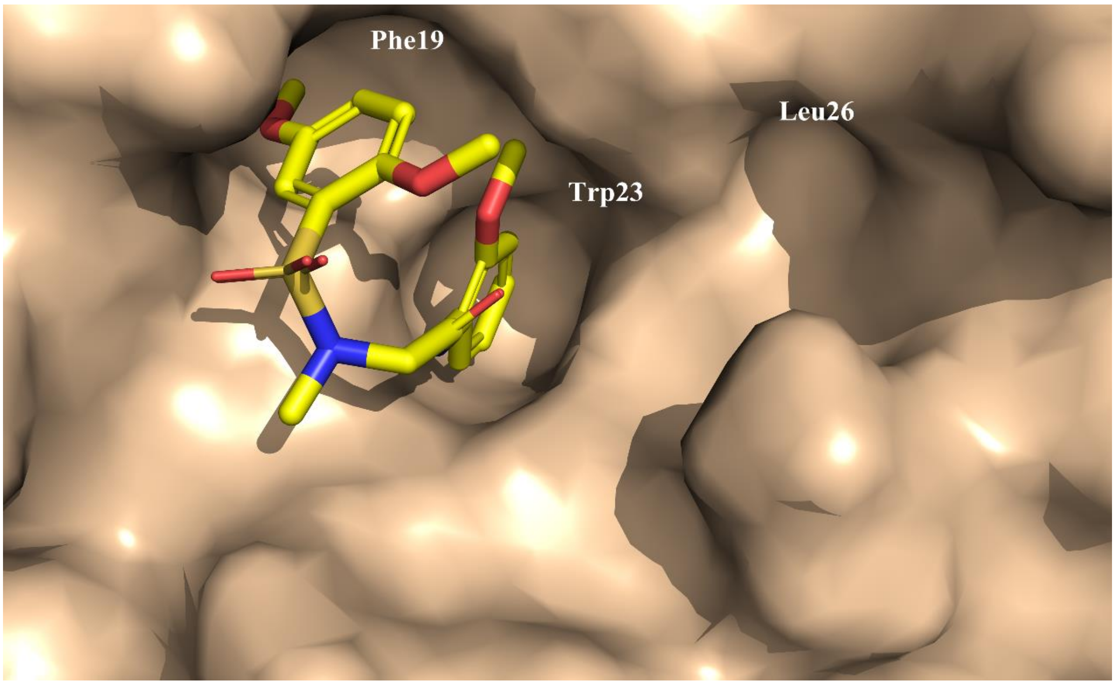

Figure S8 Predicted binding modes of **ZINC00687864** to MDM2. The protein displayed as a gray surface and its key residues were shown with labels. All compounds are shown with only backbone atoms.

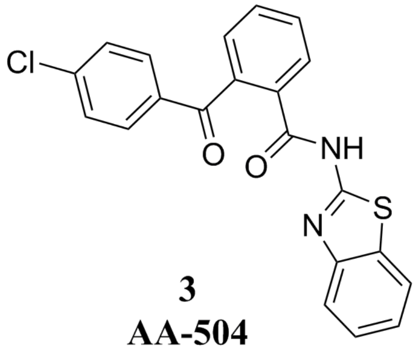

3
AA-504

Figure S9 the structure of **AA-504**

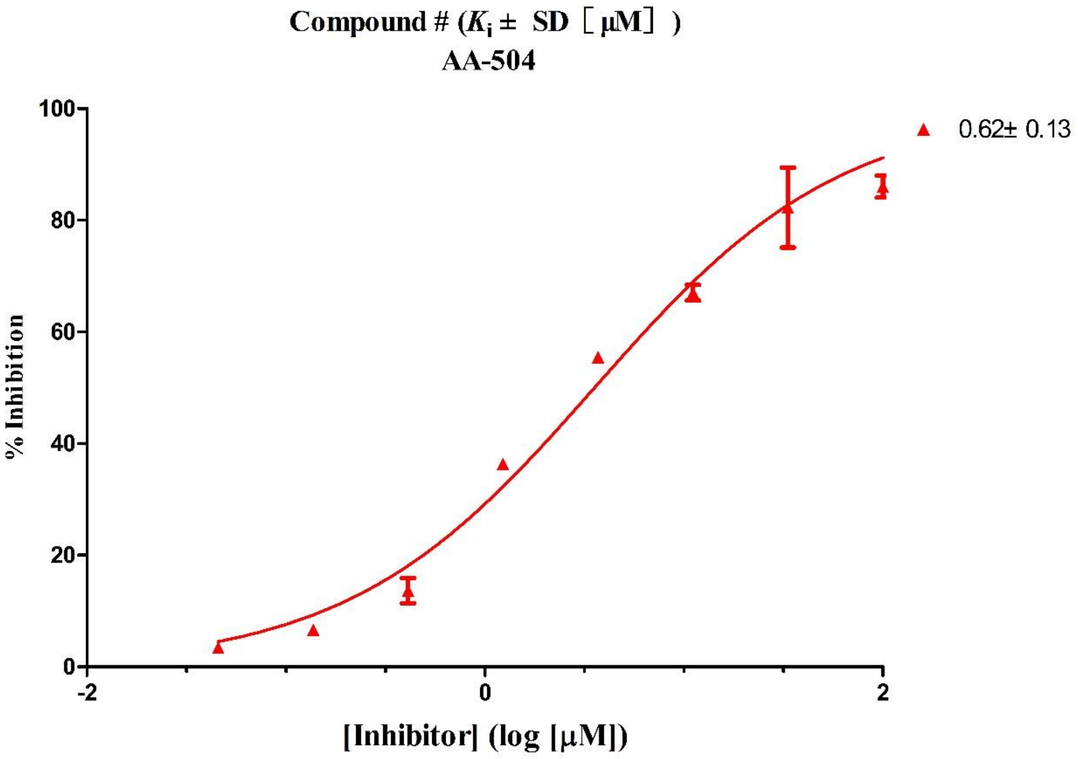

Figure S10 the curve of inhibitory of **AA-504**

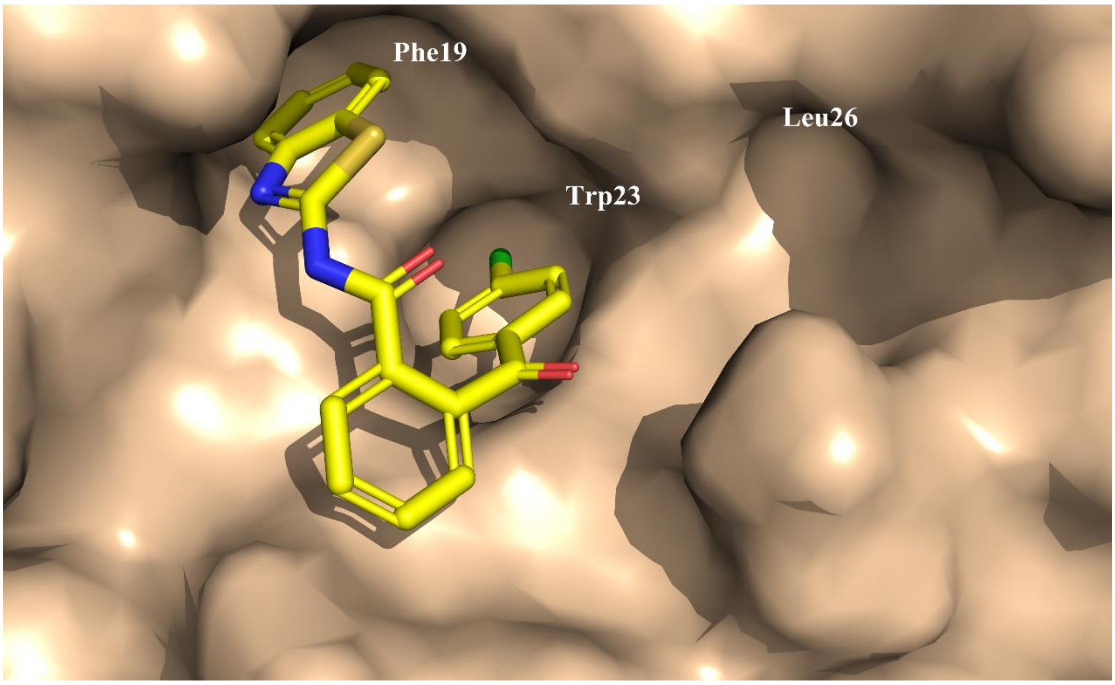

Figure S11 Predicted binding modes of compound **AA-504** to MDM2. The protein displayed as a gray surface and its key residues were shown with labels. All compounds are shown with only backbone atoms.

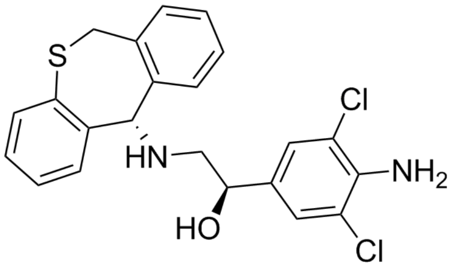

4

ZINC8385603

Figure S12 the structure of **ZINC8385603**

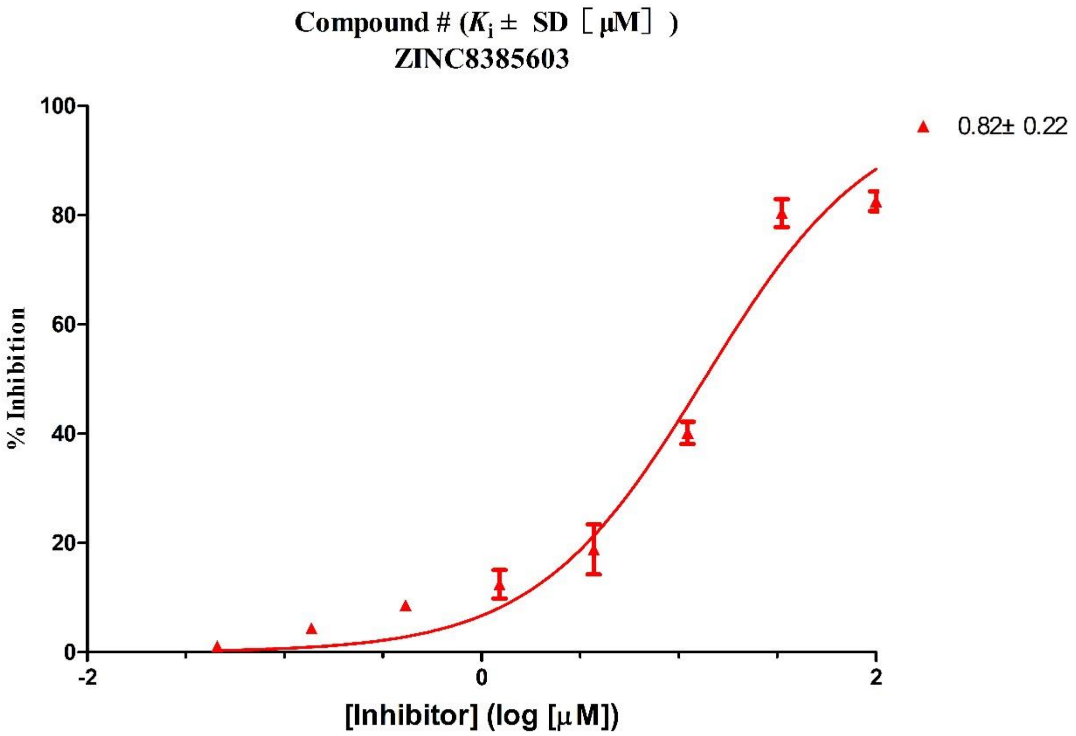

Figure S13 the curve of inhibitory of **ZINC8385603**

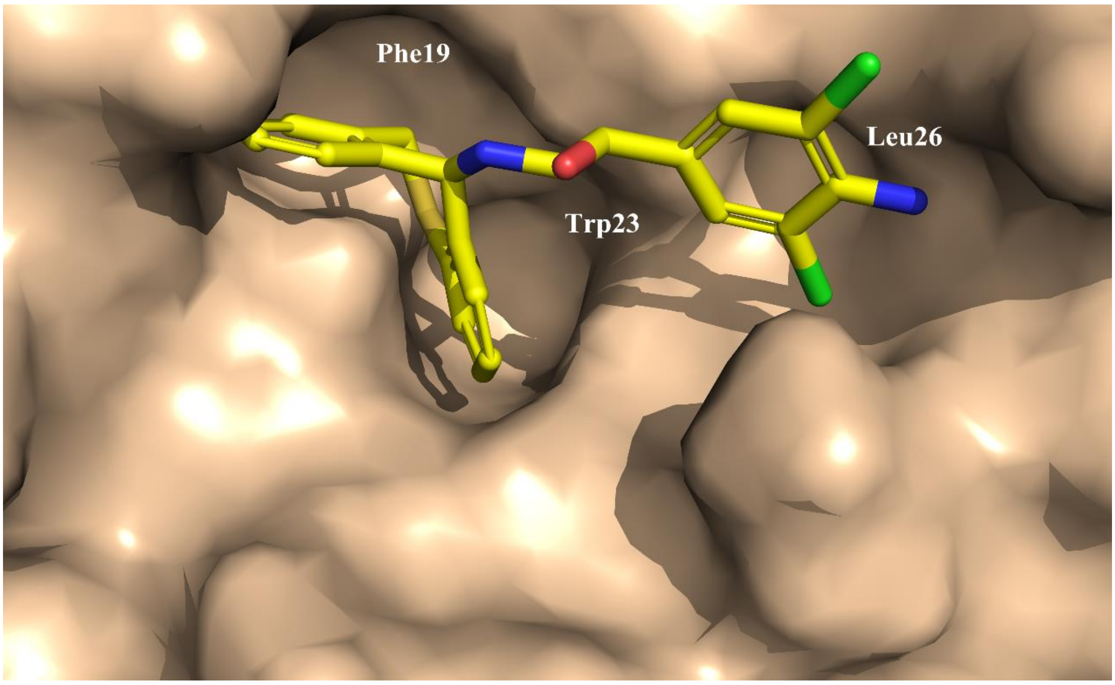

Figure S14 Predicted binding modes of **ZINC8385603** to MDM2. The protein displayed as a gray surface and its key residues were shown with labels. All compounds are shown with only backbone atoms.

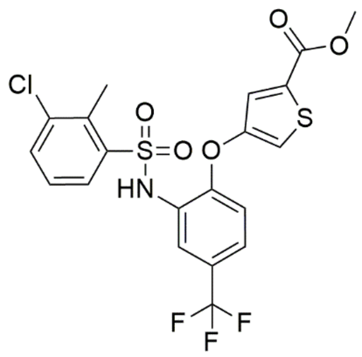

5
ZINC01027435

Figure S15 the structure of **ZINC01027435**

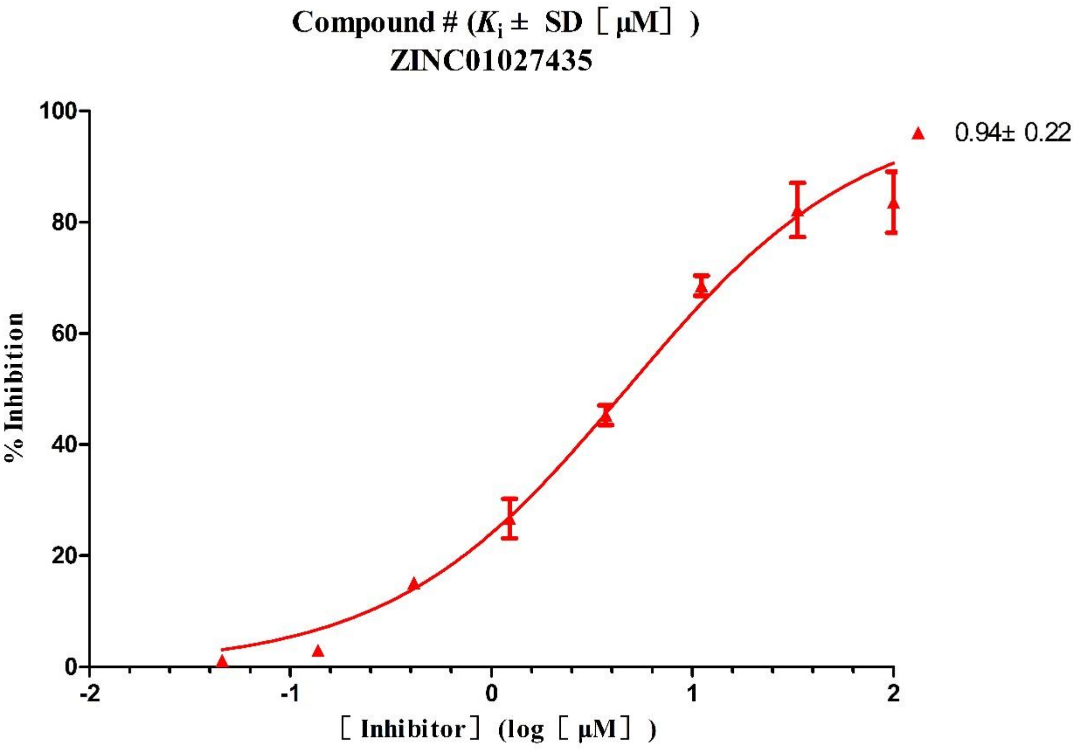

Figure S16 the curve of inhibitory of **ZINC01027435**

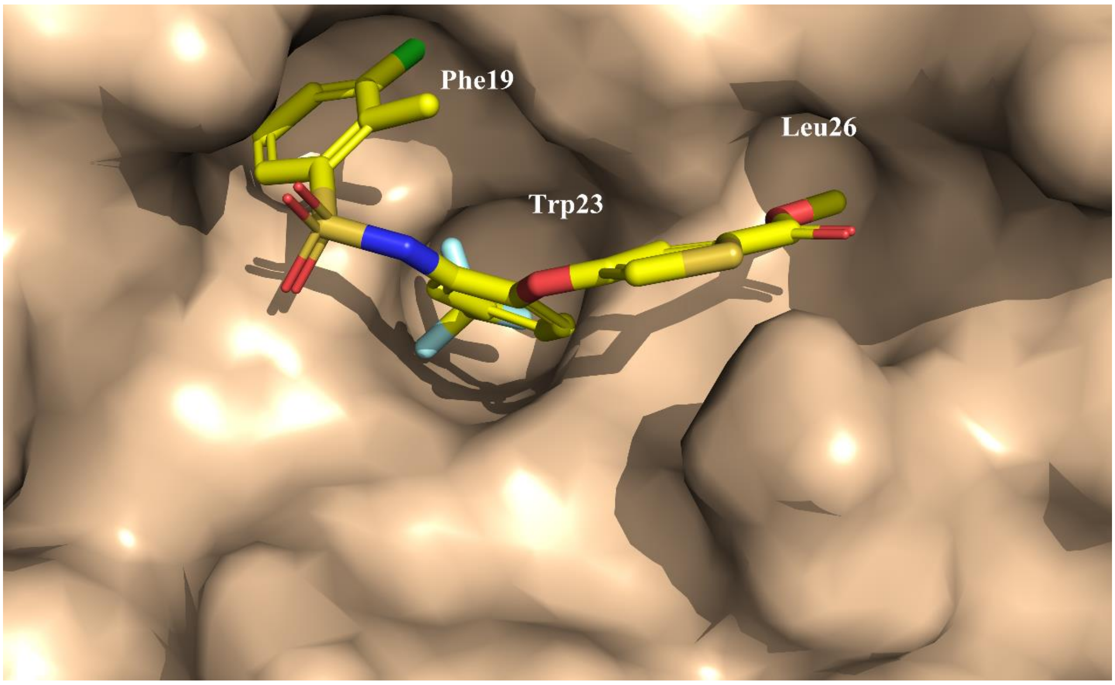

Figure S17 Predicted binding modes of **ZINC01027435** to MDM2. The protein displayed as a gray surface and its key residues were shown with labels. All compounds are shown with only backbone atoms.

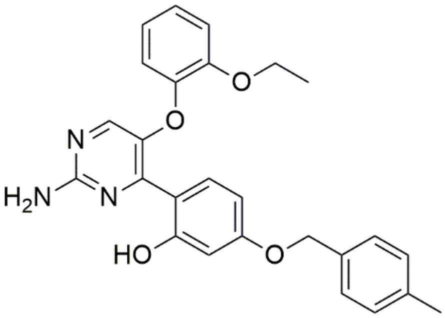

6
ZINC02331744

Figure S18 the structure of **ZINC02331744**

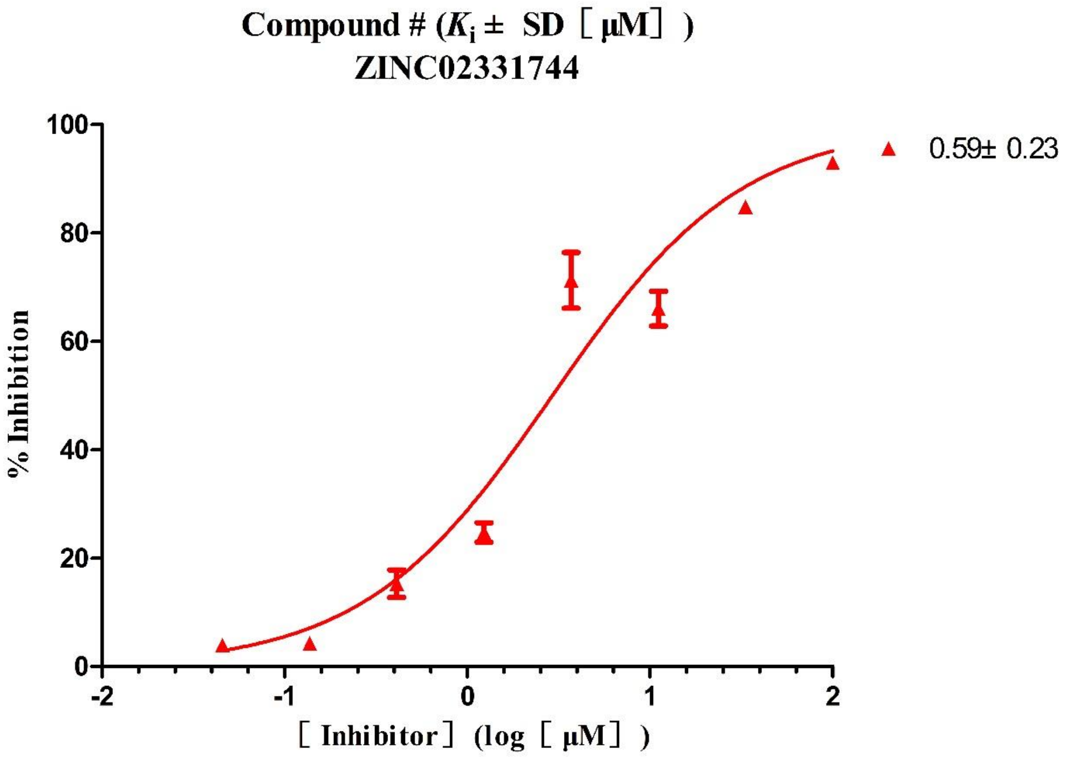

Figure S19 the curve of inhibitory of **ZINC02331744**

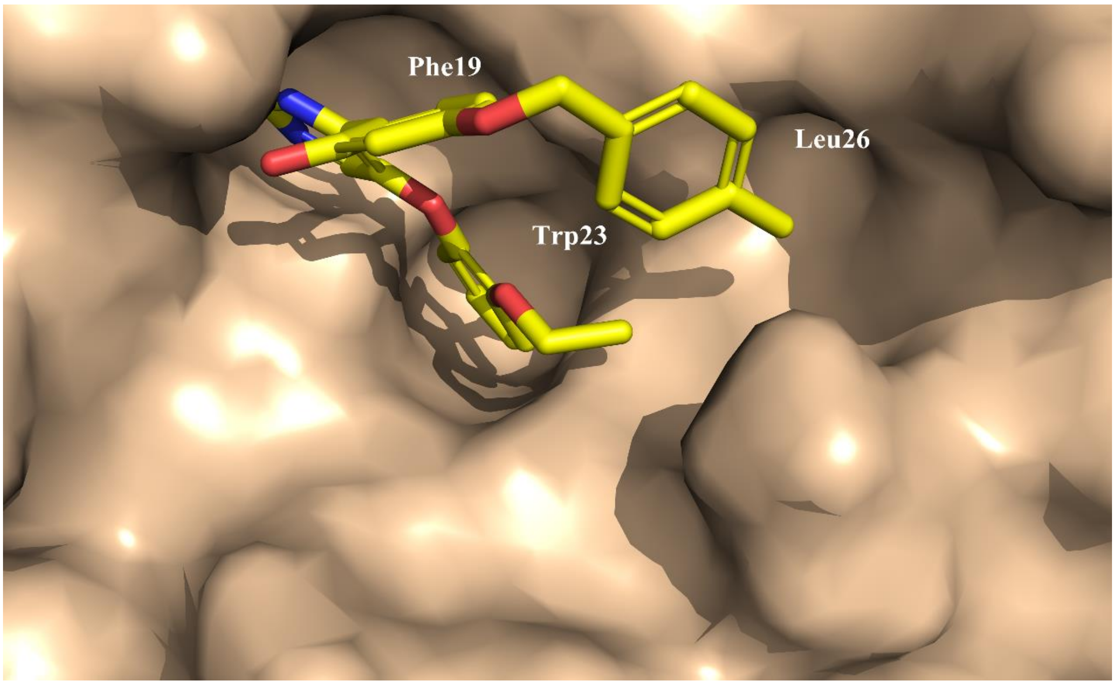

Figure S20 Predicted binding modes of **ZINC02331744** to MDM2. The protein displayed as a gray surface and its key residues were shown with labels. All compounds are shown with only backbone atoms.

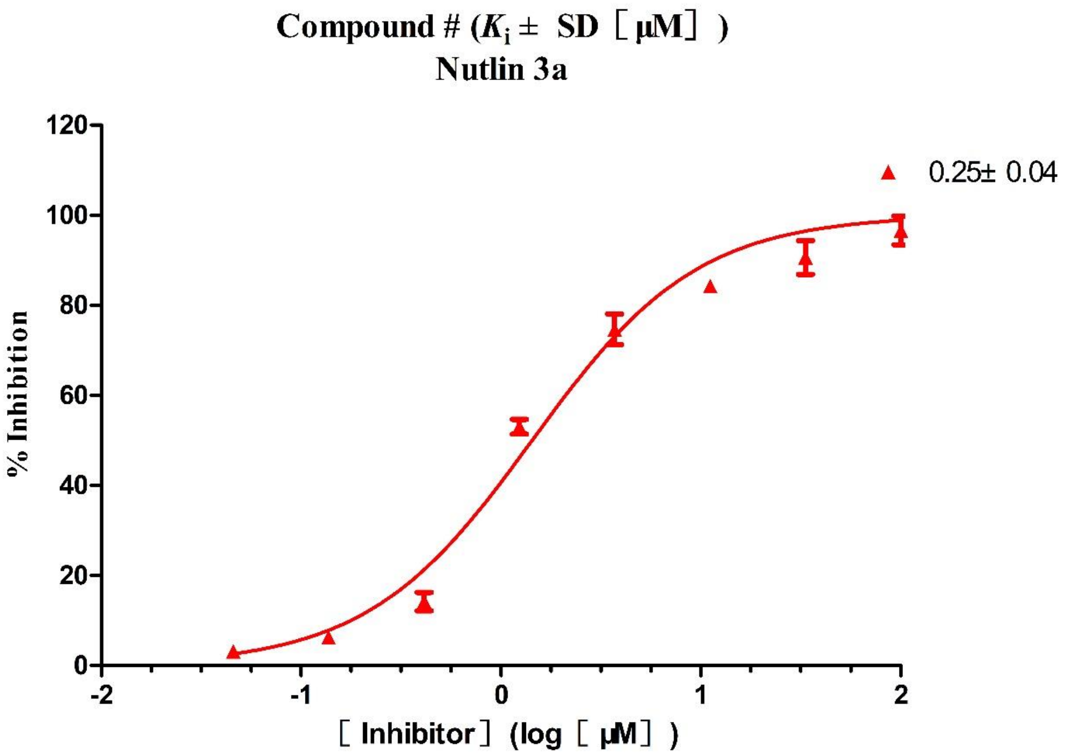

Figure S21 the curve of inhibitory of **Nutlin 3a**

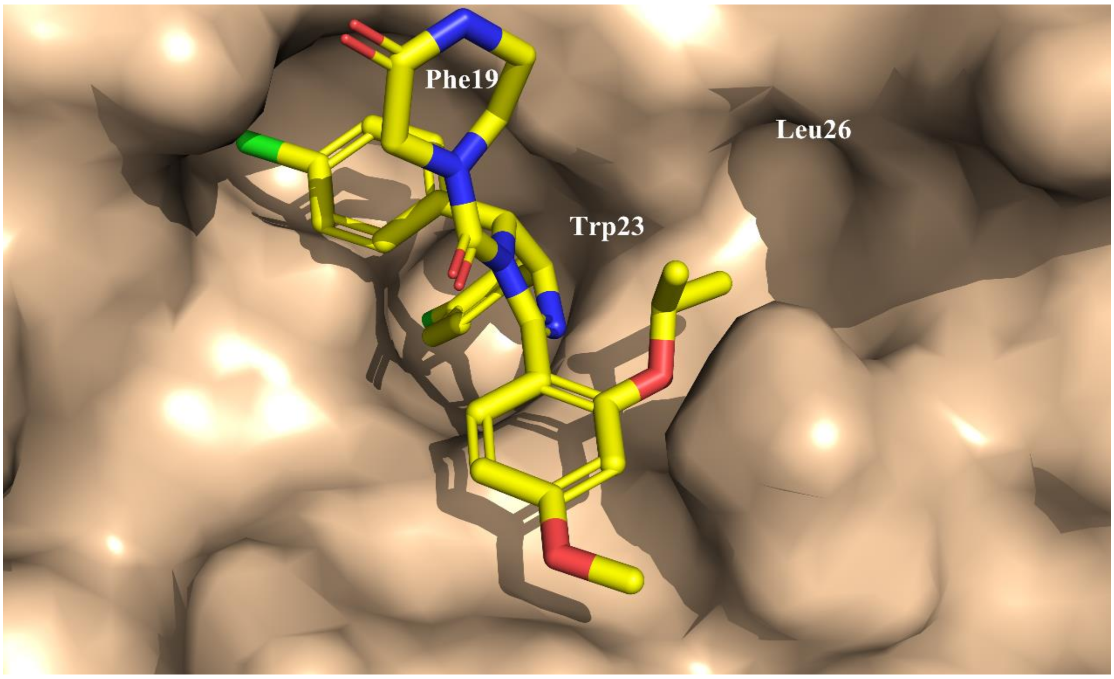

Figure S22 Predicted binding modes of **Nutlin 3a** to MDM2. The protein displayed as a gray surface and its key residues were shown with labels. All compounds are shown with only backbone atoms.

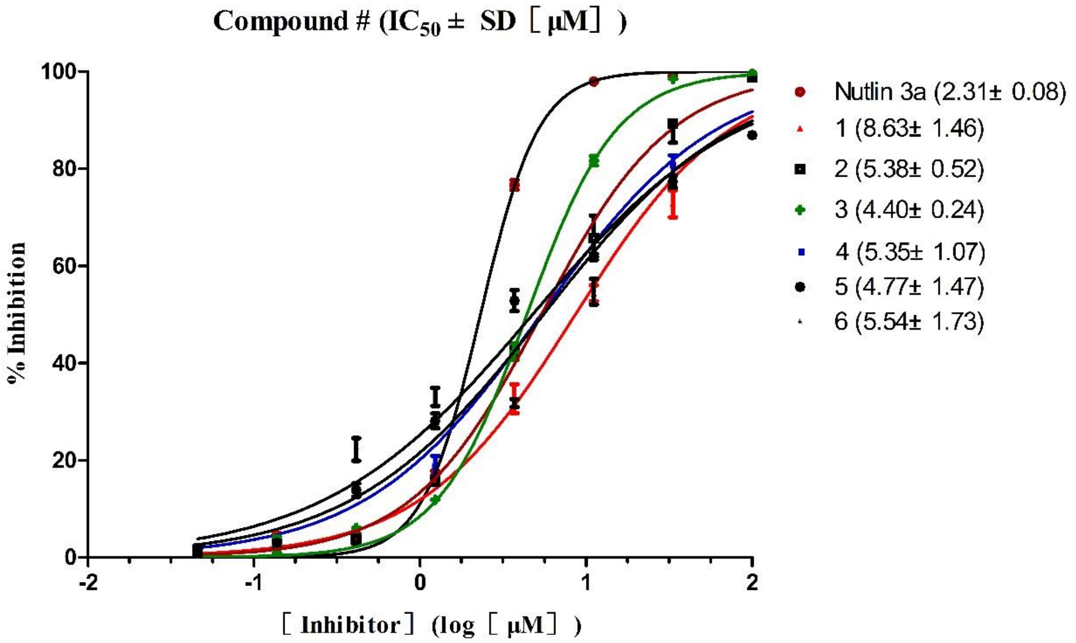

Figure S23. Curves of small-molecule inhibitors to MDM2/p53 PPI against HepG2 cell line.

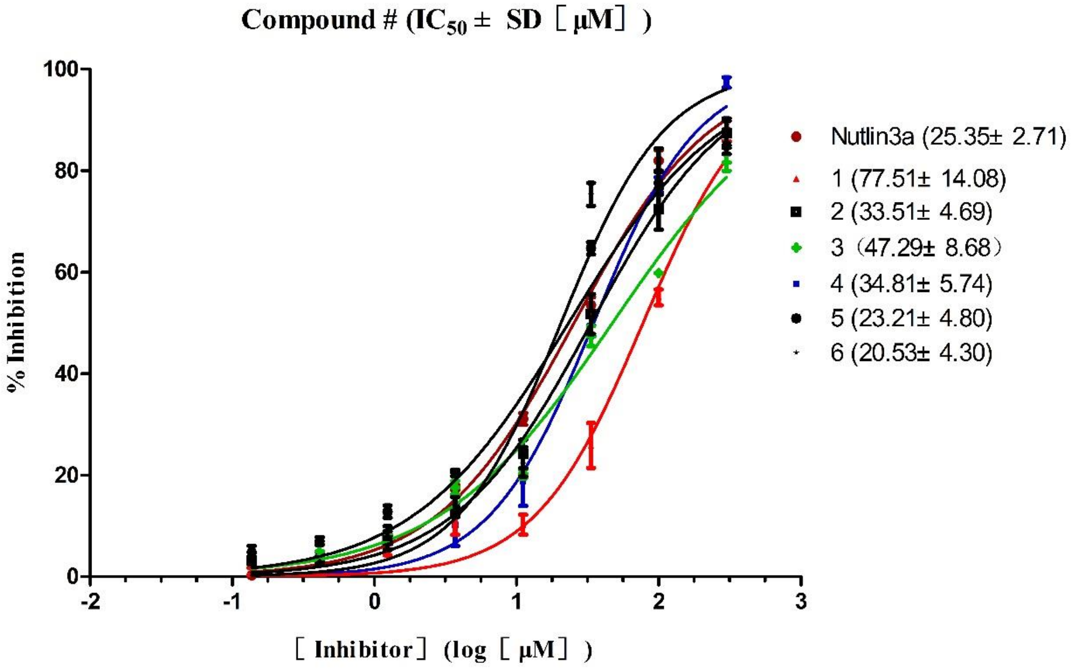

Figure S24. Curves of small-molecule inhibitors to MDM2/p53 PPI against Hep3B cell line.

REFERENCES

1. Bowman, A. L.; Nikolovska-Coleska, Z.; Zhong, H.; Wang, S.; Carlson, H. A. Small molecule inhibitors of the MDM2-p53 interaction discovered by ensemble-based receptor models. *J. Am. Chem. Soc.* **2007**, 129 (42), 12809-14.

2. Zhuang, C.; Miao, Z.; Zhu, L.; Dong, G.; Guo, Z.; Wang, S.; Zhang, Y.; Wu, Y.; Yao, J.; Sheng, C.; Zhang, W. Discovery, synthesis, and biological evaluation of orally active pyrrolidone derivatives as novel inhibitors of p53-MDM2 protein-protein interaction. *J. Med. Chem.* **2012**, 55 (22), 9630-42.

3. Popowicz, G. M.; Czarna, A.; Wolf, S.; Wang, K.; Wang, W.; Domling, A.; Holak, T. A. Structures of low molecular weight inhibitors bound to MDMX and MDM2 reveal new approaches for p53-MDMX/MDM2 antagonist drug discovery. *Cell Cycle* **2010**, 9 (6), 1104-11.
4. Nikolovska-Coleska, Z.; Wang, R.; Fang, X.; Pan, H.; Tomita, Y.; Li, P.; Roller, P. P.; Krajewski, K.; Saito, N. G.; Stuckey, J. A.; Wang, S. Development and optimization of a binding assay for the XIAP BIR3 domain using fluorescence polarization. *Anal. Biochem.* **2004**, 332 (2), 261-73.
